# Cold Plasma-Assisted Extraction of Phytochemicals: A Review

**DOI:** 10.3390/foods12173181

**Published:** 2023-08-24

**Authors:** Mahshid Heydari, Katya Carbone, Fabio Gervasi, Ehsan Parandi, Milad Rouhi, Omid Rostami, Reza Abedi-Firoozjah, Azin Kolahdouz-Nasiri, Farhad Garavand, Reza Mohammadi

**Affiliations:** 1 Student Research Committee, Department of Food Science and Technology, School of Nutrition Sciences and Food Technology, Kermanshah University of Medical Sciences, Kermanshah 6719851552, Iran; mdheydari1371@gmail.com (M.H.);; 2 CREA Research Centre for Olive, Fruit and Citrus Crops, Via di Fioranello 52, 00134 Rome, Italy; fabio.gervasi@crea.gov.it; 3 Department of Food Science & Technology, Faculty of Agricultural Engineering and Technology, University of Tehran, Karaj 3158777871, Iran; 4 Department of Food Science and Technology, School of Nutrition Sciences and Food Technology, Research Center for Environmental Determinants of Health (RCEDH), Health Institute, Kermanshah University of Medical Sciences, Kermanshah 6719851552, Iran; 5 Student Research Committee, Department of Food Science and Technology, National Nutrition and Food Technology Research Institute, Faculty of Nutrition Sciences, Food Science and Technology, Shahid Beheshti University of Medical Sciences, Tehran 1981619573, Iran; 6 Department of Food Chemistry & Technology, Teagasc Moorepark Food Research Centre, Fermoy, Co., P61 C996 Cork, Ireland

**Keywords:** green extraction, antioxidants, cold plasma, phenolic content, VOSviewer

## Abstract

In recent years, there has been growing interest in bioactive plant compounds for their beneficial effects on health and for their potential in reducing the risk of developing certain diseases such as cancer, cardiovascular diseases, and neurodegenerative disorders. The extraction techniques conventionally used to obtain these phytocompounds, however, due to the use of toxic solvents and high temperatures, tend to be supplanted by innovative and unconventional techniques, in line with the demand for environmental and economic sustainability of new chemical processes. Among non-thermal technologies, cold plasma (CP), which has been successfully used for some years in the food industry as a treatment to improve food shelf life, seems to be one of the most promising solutions in green extraction processes. CP is characterized by its low environmental impact, low cost, and better extraction yield of phytochemicals, saving time, energy, and solvents compared with other classical extraction processes. In light of these considerations, this review aims to provide an overview of the potential and critical issues related to the use of CP in the extraction of phytochemicals, particularly polyphenols and essential oils. To review the current knowledge status and future insights of CP in this sector, a bibliometric study, providing quantitative information on the research activity based on the available published scientific literature, was carried out by the VOSviewer software (v. 1.6.18). Scientometric analysis has seen an increase in scientific studies over the past two years, underlining the growing interest of the scientific community in this natural substance extraction technique. The literature studies analyzed have shown that, in general, the use of CP was able to increase the yield of essential oil and polyphenols. Furthermore, the composition of the phytoextract obtained with CP would appear to be influenced by process parameters such as intensity (power and voltage), treatment time, and the working gas used. In general, the studies analyzed showed that the best yields in terms of total polyphenols and the antioxidant and antimicrobial properties of the phytoextracts were obtained using mild process conditions and nitrogen as the working gas. The use of CP as a non-conventional extraction technique is very recent, and further studies are needed to better understand the optimal process conditions to be adopted, and above all, in-depth studies are needed to better understand the mechanisms of plasma–plant matrix interaction to verify the possibility of any side reactions that could generate, in a highly oxidative environment, potentially hazardous substances, which would limit the exploitation of this technique at the industrial level.

## 1. Introduction

In recent years, growing consumer demand for natural-based products in the personal care and well-being sectors has sparked renewed interest in medicinal and aromatic plants as sources of bioactive and functional components, especially in the wake of the recent pandemic events. Globally, the phytochemicals market is experiencing a significant positive trend lately, with a Compound Annual Growth Rate (CARG) of about 8.5%, corresponding to a trade value of about USD 7 million in 2023 [1]. Phytochemicals (term generically referring to the secondary metabolites of plants such as polyphenols) are non-nutrient bioactive components of plant origin recognized to be of great interest for their beneficial effects on human health and beyond. They include a plethora of different chemical structures that can be grouped into polyphenols, terpenoids, alkaloids, glucosinolates, etc. [2] (Table 1).

Yet, the choice of which extraction method needs to be applied to the plant matrix for their recovery is the first critical issue in maintaining their quality and biological potential unaltered. Extraction can be described as a critical transport phenomenon that transfers matrix components to the solvent [4,5,6,7], which can be carefully controlled to preserve odor, flavor, and biologically active compounds [8,9,10,11]. Table 2 shows an overview of the advantages and disadvantages of different approaches for the extraction of phytochemicals. The biological potential of natural extracts is influenced by both the technology used to obtain them and the quality of the plant matrix used [7,10,12,13]. Conventional extraction strategies to isolate bioactive compounds from plants are based on the use of organic solvents, most of which have a negative environmental impact [14,15,16,17,18]. These extraction methods, including steam distillation, hydro distillation, and liquid–solvent extraction, require high temperatures, long processing times, and at times many extraction steps, limited extraction efficiency, safety issues, and environmental concerns regarding the use of toxic solvents [19,20,21,22,23,24]. Recently, green, and non-thermal alternatives such as ultrasounds, pressurized liquids, supercritical fluid extraction, pulsed electric fields, and cold plasma (CP) have been introduced to partially overcome these problems [7,25,26,27,28]. These new methods can reduce energy consumption, process time, use of organic solvents, and loss of nutrient/nutraceutical compounds, enabling high-quality functional plant extracts [14,29,30,31].

**Figure 1:**
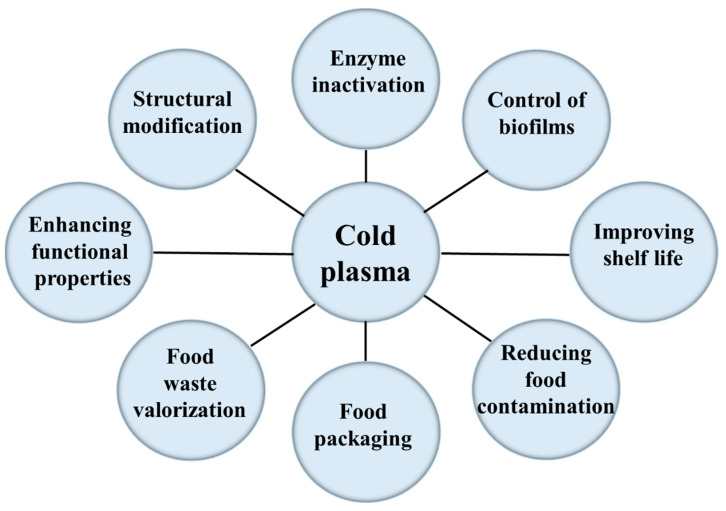
Applications of cold plasma in food-related fields.

CP is an emerging and relatively unexplored non-thermal technology with promising applications in various areas of food processing [72,73,74]. It is regarded as an innovative method, which uses highly reactive charged molecules and gaseous species to inactivate contaminating microorganisms in food. From a chemical-physical perspective, plasma represents the fourth state of matter [73,75]. When the energy of molecules in a system increases, solids turn into liquids and liquids into gases. The intermolecular configuration is altered. A further increase in the energy of gases causes all interactions to vanish, releasing positive and negative ions and causing some molecules and atoms to ionize, giving rise to plasma, which can then be defined as an ionized gas [72,73,74]. Currently, CP is used to improve the shelf life of food [75,76], reduce food contamination [77], and improve the functional properties of proteins [78]. It is also used for structural modification [79], enzyme inactivation [80], removal of toxins [81], change of food packaging characteristics [82], wastewater treatment [8,83,84], control of biofilm [85], and food waste valorization [24,86] (Figure 1). CP can also reduce the unpleasant effects of thermal treatments, such as discoloration and loss of nutrients [72,87].

In recent years, a growing number of scientific studies have highlighted the potential of using CP to increase the extraction yield of phytochemicals due to its non-thermal properties and energy efficiency [19,21,30,77,88,89], ensuring faster times, reducing the degradation of heat-sensitive substances, and achieving sophisticated extraction without impacting the environment. However, to date, most studies have focused on the microbial decontamination properties of CP, while there are few studies on the effect of CP treatment on the production of plant extracts and their phytochemical profile and relative biological potential. Thus, this work aims to review up-to-date information on the application of CP technology for the extraction of valuable plant compounds, considering its effect on (a) extraction yield, (b) total phenolic content and phytochemical profile, (c) antioxidant capacity, and (d) antimicrobial properties of the obtained extracts.

## 2. Scientometric Analysis

To obtain a general overview of international studies published up to the 24 April 2023 on the use of CP in the extraction of phytochemicals, the scientific literature was analyzed using the Scopus database (Elsevier) and the VOSviewer software (v. 1.6.18).

The Scopus search was performed (Search within Article title, Abstract, Keywords) by typing the keywords (Search documents): “cold plasma” AND “extraction”. The resulting 124 papers were examined, selecting for analysis only those documents that had all keywords typed between the title, abstract, or author keywords. This sub-selection resulted in the collection of 77 documents, which constituted the bibliographic dataset, first analyzed using the Scopus Analyze search results function (Documents by year, Documents by type, Documents by subject area, and Documents by country or territory), from which all Scopus information data analyzed with the VOSviewer software were exported.

Figure S1a shows the trend in the number of articles by year of publication, highlighting that the application of CP as an extraction technique is a very recent research topic, developed mainly in the last 10 years.

Looking at the graph in detail, it can be seen that from 2012 to 2016, the trend of publications has been constant, even the number of articles has been very low. There has been a significant increase in publications since 2017, with the highest peak reached in 2022, with 23 total articles. This value is likely to be exceeded in the current year, which has produced 12 papers up to the time of the Scopus search (April 2023), indicating that the research field has attracted even more interest in the last three years. Figure S1b also points out that CP is an innovative, start-up research topic, with 68.8% of the papers consisting of articles, while only 22.1% and 6.5% of the papers consist of reviews (17 papers) or book chapters (5 papers). Furthermore, Figure S1c shows the highest interest of the scientific community in the subject area ‘Agricultural and Biological Science’ (31.5%—52 articles), with the highest number of studies concerning the use of CP for the extraction of bioactive compounds from various plant/agricultural matrices published in journals belonging to the subject area “Biochemistry, Genetics, and Molecular Biology” (7.9%—13 articles). The second and third areas of interest are represented by “Engineering” (12.7%—21 documents) and “Chemical Engineering” (10.9%—18 documents), underscoring the engineering effort required to develop CP technology for chemical extraction. The bar graph shown in Figure S1d shows the number of papers for each country/territory and, interestingly, highlights that the use of CP as an extractive technique has so far attracted the interest of mainly three nations, all from Asia, with China being the top country in terms of related scientific production (21 papers), followed by Iran (13 papers) and India (12 papers).To explore the knowledge structure in this field of research, the extracted bibliographic dataset was analyzed with VOSviewer to calculate and display a map of the keyword co-occurrence network. From the dataset, the software extracted 974 total keywords, of which 39 exceeded the predefined minimum threshold of 5 occurrences. The keyword “article” was manually excluded from the final calculation to avoid any bias in the topology of the graph. All other software settings were left as default.

**Figure 2:**
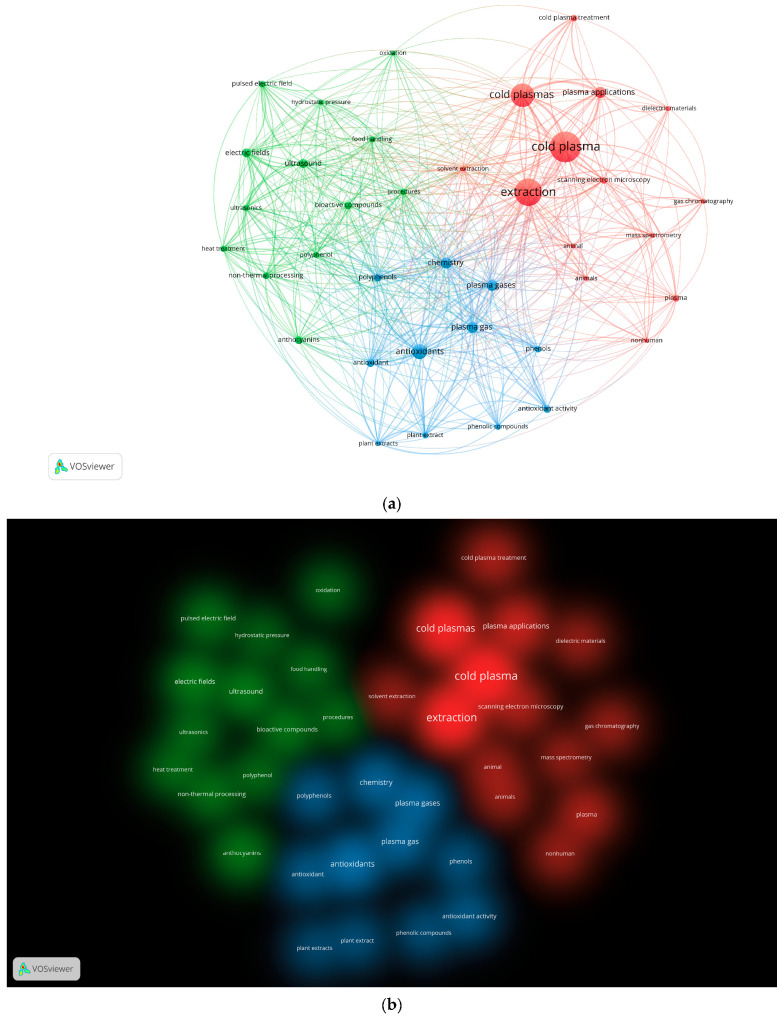
(**a**) Network Visualization of the keywords co-occurrence network map. (**b**) Density visualization (Cluster density setting). Each density representing a cluster has a different color (CS_1: red; CS_2: green; CS_3: light blue). All the visualizations were generated by the VOSViewer software from the search results in Scopus (performed on 24 April 2023) after the search: “cold plasma” AND “extraction” followed by the sub-selection of the dataset.

The Network Visualization in VOSviewer shows the keyword co-occurrence network map, which graphically represents the keywords extracted from the bibliographical dataset as points or nodes, and the co-occurrence of two keywords into the same publications as a line or link. The larger the area of a node, the higher the absolute number of occurrences of a keyword in the dataset. Nodes proximity is directly related to keywords co-occurrence: the closer two nodes are, the more the two keywords are related by similar research publications. VOSviewer identified 3 clusters (see Table S1 for cluster composition), each highlighted by a different color (red, green, light blue) in the Network Visualization map and by the same colors in the Density Visualization map (Cluster density; CS) (Figure 2a,b).

The network map shows a general topology of highly interconnected nodes among the three clusters, with the absence of any overlap and the absence of a single central node, but with two nearby central nodes represented by the keywords ‘cold plasma’ (30 occurrences) and ‘extraction’ (27 occurrences), both in cluster 1 (CS_1). The small size of all nodes (low number of total occurrences of each keyword) and the low number of nodes that make up the graph, together with the absence of areas of overlap between clusters, confirm that the use of CP for chemical extraction is a very young field of research with strong growth potential for the future. CS_1 has “cold plasma” as the most recurrent keyword in the cluster (n. of occurrences 30), which is related to almost all nodes (keywords) in the entire graph. CS_1 is composed of 14 keywords extracted from publications on the characterization of the effects of CP technology on complex matrices, subsequently used for chemical extraction at the microscopical and chemical level: “extraction”, “plasma application”, “scanning electron microscopy”, “gas chromatography”, and “mass spectrometry”. In CS_2, the ‘electric fields’ keyword is the most recurrent (9) out of a total of 13 keywords, all related to the testing of different extraction technologies coupled with CP for different applications: “pulsed electric field”, “hydrostatic pressure”, “ultrasound”, “ultrasonics”, “non-thermal processing”, and “food handling”. CS_3, in which the most recurrent keyword is ‘antioxidants’ with 15 total occurrences in the analyzed documents, is composed of 11 nodes, graphically representing research on the use of plasma gas for the chemical extraction of phenolic compounds and antioxidants from plant matrices: “plasma gas”, “polyphenols”, “phenolic compounds”, “phenols”, “plant extract”, and “antioxidant activity”.

**Figure 3:**
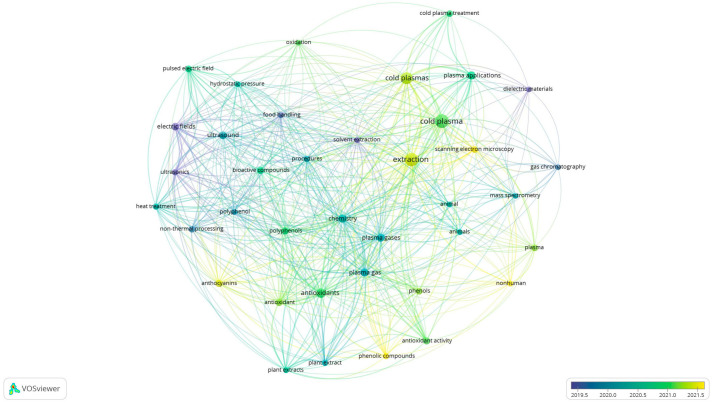
Overlay visualization of the keyword co-occurrence network map which highlights the average year of publication of the keywords in the map (AYP). All the visualizations were generated by the VOSViewer software from the search results in Scopus (performed on 24 April 2023) after the search: “cold plasma” AND “extraction” followed by the sub-selection of the dataset.

The overlay view of the network map drawn by VOSviewer (Figure 3) shows, for each keyword, the average year of publication (AYP), with a color scale ranging from dark blue, for older publications, to bright yellow, for more recent ones.

The analysis conducted suggests that the earliest applications of CP for the extraction of phytocompounds were in ‘food processing’ using ‘ultrasound’ and/or ‘electric fields’. In contrast, the results highlight very recent applications studies on cold plasma for the ‘extraction’ of ‘phenolic compounds’ and ‘anthocyanins’ from complex matrices, analyzing the effects of this technology using ‘scanning electron microscopy’.

## 3. Principles, Types, and Sources of Cold Plasma Production

**Figure 4:**
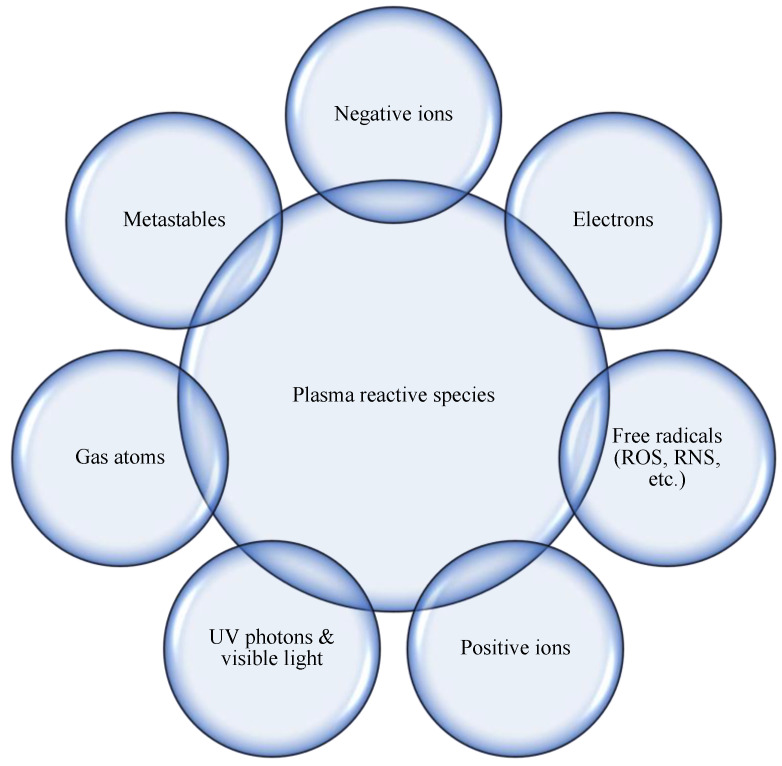
Plasma reactive species.

Plasma is considered the fourth state of the matter. It is a partially or fully ionized gas, macroscopically neutral, in which the species that can be identified are the molecules of the gas, fragments of the same (atoms, positive and negative ions, and radicals), and reaction products between all the species present [90] (Figure 4); these may be in different states of excitation depending on how the energy is distributed within the system.

Any gas at a temperature above 0 °K contains a certain concentration of charged species (electrons and ions), but it is only considered a plasma if the concentration of charged species is such as to affect its motion. Any form of energy, including UV and gamma radiations, electrical energy, and electromagnetic radiation, has the potential to generate plasma. Plasma can be classified based on temperature (hot plasma or cold plasma), pressure (low, atmospheric, or high pressure), working gas (air, oxygen, argon, or helium), and mode of production. Non-thermal atmospheric pressure plasma (non-equilibrium plasma) is commonly described as CP, in which the temperature of the ions and neutral atoms is significantly lower than that of the electrons. Thus, it is characterized by a non-uniform distribution of energy among the constituent particles. Since the ions and neutral atoms remain relatively cold, CP can be successfully used with heat-sensitive compounds.

**Figure 5:**
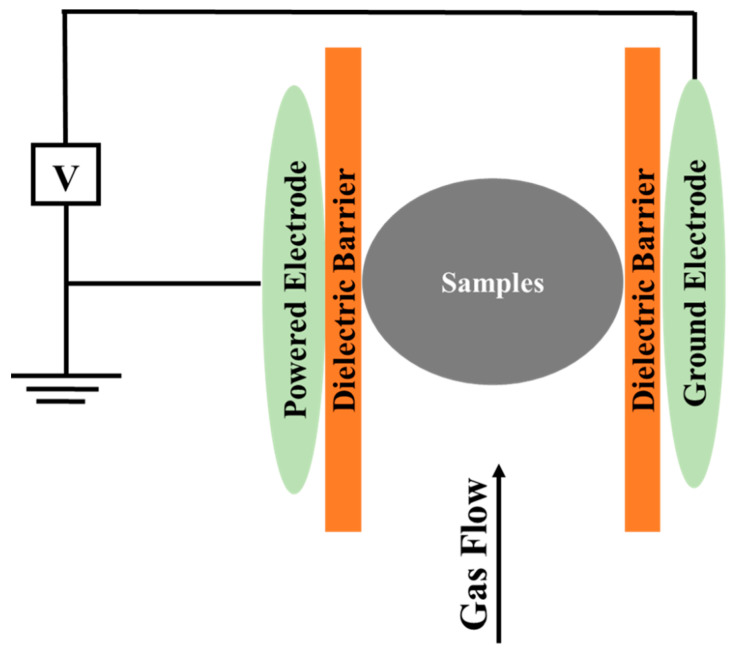
Dielectric barrier discharge system to generate cold plasma.

CP emits light with wavelengths in both the visible and ultraviolet spectral regions. In addition to the emission of UV radiation (wavelength range: 100–380 nm), an important property of low-temperature plasma is the presence of high-energy, highly reactive electrons, which cause several chemical and physical processes such as oxidation, the excitation of atoms and molecules, the production of free radicals, and UV photons that can decompose covalent bonds and produce many chemical reactions, such as surface etching (creation of pores/tissue damage), depolymerization (formation of new compounds through the breakdown of cell wall polysaccharides), and cross-linking (cleavage of C-OH polymeric chains for adding new C-O-C bond by eliminating water) [72,87]. In addition, UV photons can increase the activity of specific enzymes such as phenylalanine ammonia-lyase, thereby increasing the amount of total phenols extracted from the plant matrix [91]. Plasma can be generated artificially by supplying a gas with sufficiently high energy employing lasers, shock waves, electric arcs, and electric and magnetic fields, i.e., by applying energy to a gas in such a way as to reorganize the electronic structure of species (atoms, molecules) and produce excited species and ions. One of the most common ways to artificially create and maintain plasma is by using an electric discharge in a gas. Other ways include the use of jet plasma (a jet plasma system is connected to a power source, which induces ionization in the surrounding gas) and microwave plasma (plasma torches with two dielectric tubes that let the gas pass through). In the case of CP, so-called non-thermal discharges are used [30,72,92]. The two main types of non-thermal discharge at atmospheric pressure are corona discharge and dielectric barrier discharge (DBD) [93]. DBD is the commonly used system for the extraction of bioactive compounds from plant matrices [20,72,88,94]. In a DBD system, an electrical discharge is generated by two electrodes that are separated by a dielectric layer (a material with high electrical resistance) (Figure 5). It operates at approximately atmospheric pressure (0.1–1 atm), at frequencies up to 104 Hz, and alternating voltages up to 100 kV [72].

The most important parameters influencing the CP process are the plasma generation voltage or power (higher power increases the electron density, generating more reactive species capable of interacting with the matrix to be treated), the amount of material to be treated (efficiency decreases as the amount increases), the duration of exposure (time), the gas pressure (affects the rate of plasma volatilization), the gas flow rate (higher gas flow rate can improve treatment efficiency), and the type of feed gas used [30,92]. The degree to which reactive species are produced depends crucially on the gas used to generate the plasma (working gas). Plasma gases, such as oxygen, nitrogen, and dry air, are commonly employed in the food processing industry [72,87,89]. However, gas mixtures such as He/N_2_, He/O_2_, N_2_/N_2_O, Ar/O_2_, etc., can also be used.

Another important parameter influencing the effectiveness of plasma treatment in extracting phytochemicals is the frequency of plasma generation, due to the inverse relationship between the frequency and the activity of certain crucial enzymes such as peroxidase and polyphenol oxidase [95].

## 4. Mode of Action of Cold Plasma on the Plant Matrix and Its Critical Issues

The extraction yield of plant components can be affected by several factors [30,76,92], including the level of disruption of the cell wall, CP process parameters (type of extraction method), plant species, material surfaces, and composition of the bioactive compounds that occurred in the plant (Table 3). It has been demonstrated that CP treatment can cause changes in the surface physical properties of the plant matrix, with the formation of cracks and depressions on its surface that allow better outward release of the compounds of interest, thus increasing the extraction yield. In addition, CP appears to be able to increase the hydrophilicity of the matrix surface through the degradation of the cuticular layer, thereby facilitating the diffusion of internal molecules to the solvent and consequently increasing the extractability of hydrophilic compounds, such as phenols. These mechanisms are attributable to the action of plasma-generated reactive species, mainly ROS and RNS, which can modify the surfaces of materials and change their functional groups, as stated previously.

**Figure 6:**
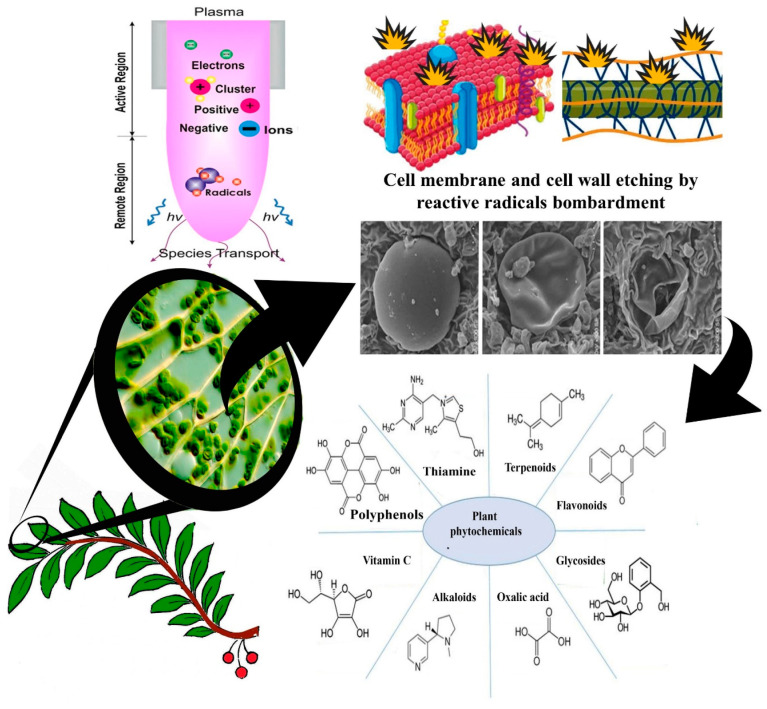
Possible release mechanisms of phytochemicals through the interaction between cold plasma and plant matrix.

Figure 6 shows the possible mechanism of action of CP responsible for the release of bioactive substances from plant matrices.

**Figure 7:**
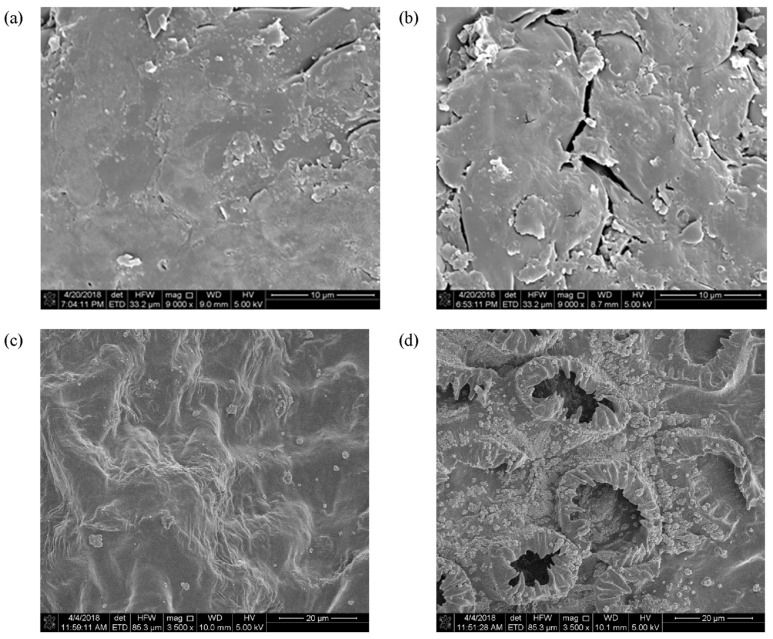
Scanning electron microscope images of (**a**) untreated dry fenugreek seeds, (**b**) HVACP-treated dry fenugreek seeds, (**c**) untreated soaked fenugreek seeds, and (**d**) HVACP-treated soaked fenugreek seeds. Adapted with permission from Ref. [112], 2020, Elsevier.

This mechanism is supported by several findings. Rashid et al. (2020) observed that the extraction yield of galactomannan from fenugreek increased before (crushed dry seeds) and after (crushed seeds soaked in extraction solutions) treatment with high voltage cold atmospheric plasma (HVACP) by 67% and 122%, respectively, in a statistically significant manner. They concluded that the increase in dry extract yield could be due to HVACP-induced changes in the surface morphology of fenugreek seeds. Exposure to plasma active species resulted in specific fragments and cracks in the epidermal structure of the seeds (Figure 7b,d), which were able to retain and absorb the extractive solution more effectively than the control, which showed a smooth, intact surface on the microscopic investigation (Figure 7a,c) [112].

**Figure 8:**
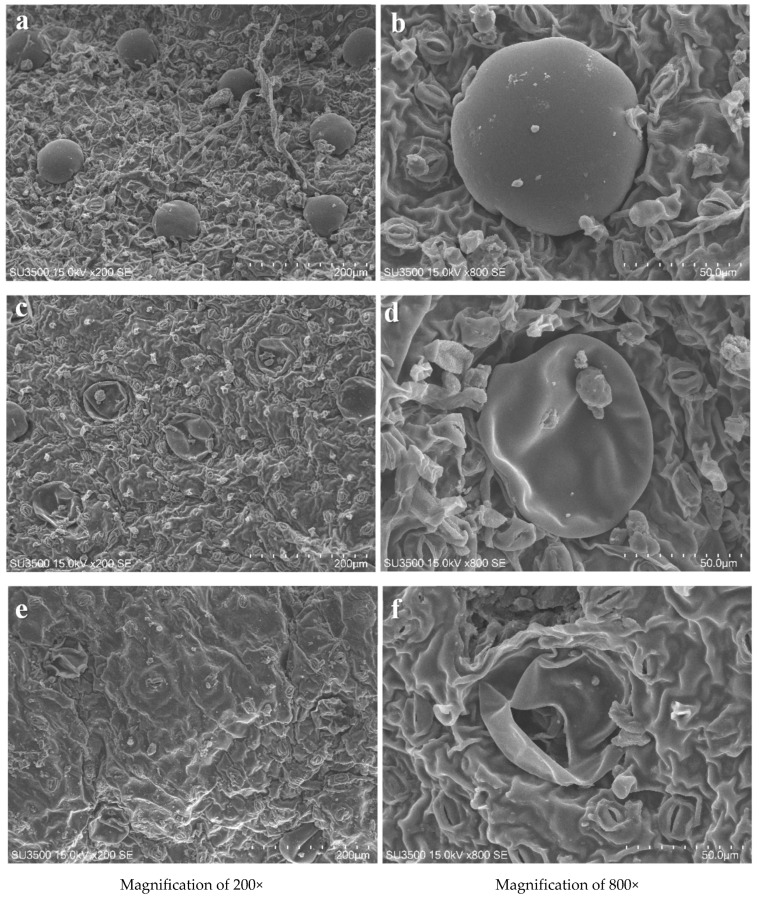
Scanning electron microscope images of (**a**,**b**) Control (spearmint leaf), (**c**,**d**) CP-treated leaf (15 kV, 5 min), (**e**,**f**) CP-treated leaf treated leaf (23 kV, 17 min) at different magnifications. Adapted with permission from Ref. [115], 2021, Elsevier.

Ebadi et al. (2019) showed that the yield of lemon verbena leaf essential oil was improved by the low-pressure cold plasma (LPCP) treatment [118]. This result was confirmed by other researchers’ observations. Kodama et al. (2014) found that plasma treatment increased the essential oil yield of lemon peel [119], while Rezaei et al. (2021) reported that the extraction efficiency of essential oil from fennel seeds (*Foeniculum vulgare* Mill.) and mint leaves (*Mentha spicata* L.) can be influenced by DBD CP treatment followed by hydrodistillation [115]. In particular, they observed a higher extraction yield with a 15 kV plasma treatment applied for 5 min (1.83 (% *v*/*w*)) than with a 23 kV treatment applied for 17 min (1.81 (% *v*/*w*)). According to Figure 8, CP disrupted the structural integrity of the seed coat, disrupting the surface oil glands and creating permeability during distillation, which resulted in a higher oil extraction yield. In contrast, intense treatments, due to prolonged plasma exposure, adversely affected the glands, producing essential oil vapors and leading to a decrease in yield. The same authors, [113], in a previous paper, showed that the application of CP treatment was able to increase the oil yield extracted from *Camelina sativa* seeds by 31.5%, correlating the results obtained to the more intense and higher treatment and processing time used (18 kV and 16 min), which could further damage the cell wall [113,123]. Similarly, Afshar et al. (2022) indicated that the extraction of sunflower and sesame oil seeds by non-thermal plasma in oxygen and nitrogen atmospheres increased the extraction yield through wall degradation of the seed cell [114,124]. Also, Pragna et al. (2019) and Kodama and Sekiguchi (2015) observed a similar effect of DBD CP-assisted hydrodistillation on the enhancement of lemon peel extraction yield [116,119].

In addition, according to Fernandes and Rodrigues (2021), CP can affect the final polyphenol content and profile of the plant extract, either positively or negatively, through other different mechanisms in addition to those described so far: (i) depolymerization of tannins, and (ii) oxidation of phenolic structures [90]. Lastly, it has been shown that CP treatment can in some cases produce a stress-induced accumulation of phenolic antioxidants, thereby increasing the extraction yield by acting on the regulation of gene expression and the activity of crucial enzymes of the phenylpropanoid pathway [90]. Conversely, one of the main critical issues related to the mechanisms of interaction between plasma and plant matrix is the possible generation, within the extract, of reactive oxygen and nitrogen species that are harmful to human health, such as hydrogen peroxide, which is capable of exerting antimicrobial and cytotoxic activity [125], or degradation compounds resulting from interactions between the reactive species and plant matrix nutrients (i.e., proteins, lipids, and carbohydrates) [126] such as malondialdehyde or 4-hydroxynonenal from unsaturated fatty acids [127].

An important prerequisite for the applicability of a plant extract, whether in the food, herbal, or cosmetic industries, is safety. In the case of plasma, in particular, the use of which generates harmful reactive species, knowledge of the potential threats from its use is even more important.

In this regard, Wende et al. (2018) pointed out, in their review of potential risks from the use of plasma in clinical applications, that the plasma sources considered were safe biologically, chemically, and physically [128].

However, if we go outside the clinical field, to date there is very little research on the safety of plasma-treated foods for human or animal consumption, while, as far as we know, there are even fewer studies in the literature on the toxic effects of phytoextracts obtained by CP.

Mehta et al. (2022) reported no toxicity towards HepG2 cells when using plasma polyphenol extracts from rice and corn at concentrations under 250 µg/mL [129].

Conversely, Heslin et al. (2020) reported contrasting effects using lettuce broth treated with plasma for different times in in vitro and in vivo experiments on *Galleria mellonella* larvae. The authors observed low cytotoxic effects in vitro and acute toxicity of the broth treated for 5 min in vivo, emphasizing the need to evaluate plasma-treated products both in isolation and in the context of a biological system and highlighting the need for detailed exposure studies and standardized evaluation procedures [130]. Los et al. (2020) reported that the biochemistry and cytotoxic potential of food models changed more when the plasma-treated sample was in liquid (aqueous wheat extract) rather than solid (whole wheat kernels) form and that the reduction in cell growth also depended on the parameters of the plasma treatment. As in other studies, the authors emphasized increased cytotoxicity when samples were exposed for long periods to plasma treatment (up to 20 min) and when cell cultures were treated with high concentrations of the test solutions (up to 10%) [131]. These results are very interesting because they highlight the possibility of low risk in the use of plasma as an extractive technique for the preparation of phytochemical extracts from a solid plant matrix. However, the lack of in-depth studies on the variation in the chemical and biochemical composition of the final extracts obtained with this technology does not allow the technique to be defined as safe for the preparation of phytochemical extracts to date, but opens up a very exciting field of research for scholars in the field.

## 5. Influence of Cold Plasma on the Phytochemical Profile and Biological Activity of Plant Extracts

### 5.1. Phytochemical Profile

**Figure 9:**
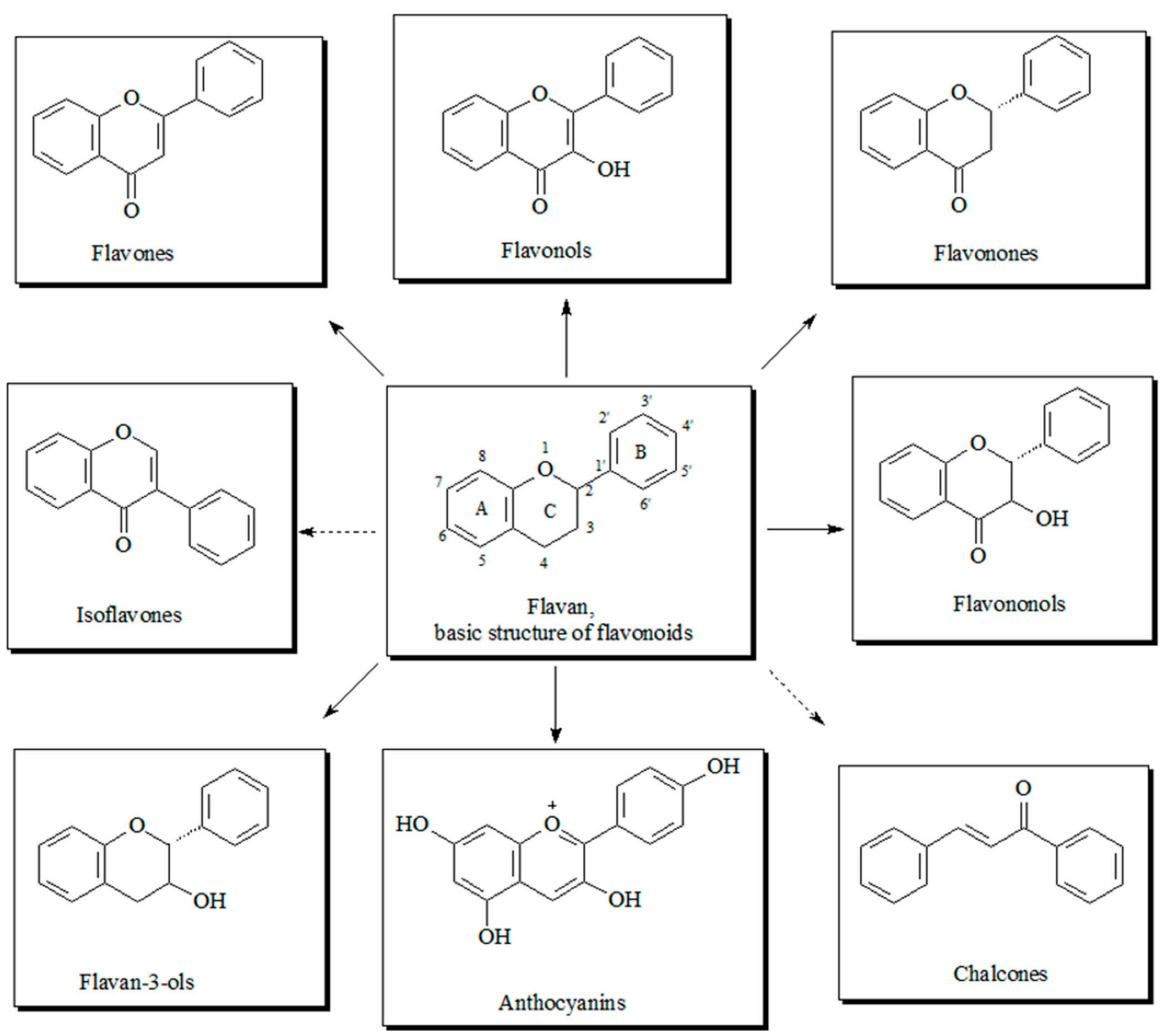
Chemical structures of the most representative polyphenol compounds.

From a chemical point of view, the term ‘phenolic’ or ‘polyphenol’ can be defined as a substance that possesses one or more aromatic ring and various functional derivatives such as esters, methyl ethers, glycosides, etc. [132] (Figure 9). In addition, most phenolic compounds possess two or more hydroxyl groups, which contribute to their potential hydrogen binding with other compounds [132].

Phenolic compounds are positively correlated with the nutraceutical and sensory quality of fresh and processed plant foods [133]. They possess a broad spectrum of biological properties such as antioxidant, antiproliferative, and anti-inflammatory (Table 1), making them excellent health-promoting compounds for the food industry [134,135,136].

Several studies have shown how CP treatment can affect the extraction of these compounds, by increasing the total polyphenol content (TPC) in the obtained plant extracts. Ahmadian et al. (2023) demonstrated that ultrasounds coupled with cold atmospheric plasma (CAP), as a pre-treatment, improved the extraction of phenolic components from *Hyssopus officinalis* L. (Hyssop) by about 22% compared with ultrasound alone [96]. Phenolic components increased in connection with cell membrane destruction by active chemical species, UV photons, and charged particles generated from CP, simplifying the extraction of phenolic compounds from the plant matrix. Also, CP supplied enough energy to break phenolic covalent bonds within polysaccharides (plant cell walls). Moreover, nitrogen plasma-pretreated samples exhibited a higher TPC than the other samples (air plasma and conventional solvent extraction). This was probably due to the breakdown of the aromatic rings of phenolic compounds by molecular ozone, which caused their destruction. However, a longer pre-treatment time with CP can create more reactive components, providing sufficient energy to separate and release the bound phenols [88,94,96,99]. Although CP-generated ozone can affect the properties of plant extracts, it is very unstable to remain in the final product [137,138,139]. Bao et al. (2020) tested a high-voltage atmospheric cold plasma (HVACP) pretreatment using different working gases (air, Ar, He, and N_2_) to evaluate the extraction of phenolics from tomato pomace. According to the main results, samples treated with He and N_2_ plasma showed significantly higher extraction yields than untreated samples. In contrast, argon and air plasma treatments showed no significant differences compared with the control. Eight phenolic compounds (such as phenolic acids and flavonoids) were also identified and confirmed by liquid chromatography mass-spectroscopy (LC-MS). CP treatments showed no effect on the concentrations of trans-ferulic acid, gallic acid, rutin, and isoquercetin, while concentrations of caffeic acid, chlorogenic acid, quercetin, and naringenin increased depending on the working gas. Additionally, the extraction rate of flavonoids was more significant than that of phenolic acids, since the release of bound flavonoids required less energy [88].

In a similar study, Bao et al. (2020) investigated the effect of HVACP on the extraction of phenolics and anthocyanins from grape pomace. Treatment with CP (5 and 15 min) significantly increased the extraction of phenols, while the sample treated for 10 min showed no significant differences compared with the control. Similarly to the phenol content, the anthocyanin content increased after treatment with CP (processing time: 5 and 15 min) compared with the untreated sample; however, the authors noted no significant difference from the control for samples treated for 10 min with CP. In the analyzed samples, anthocyanins, quercetin, and phenolic acids (such as protocatechuic and gallic acids) were identified by ultra-performance liquid chromatography (UPLC). About these compounds specifically, after treatment with CP for 5, 10, and 15 min, the results showed higher quercetin concentrations than in the control, while CP treatments lasting 5 and 15 min were able to produce increased anthocyanidin concentrations in the respective extracts. The authors also reported that, under the experimental conditions tested, flavonoids were more extractable than phenolic acids [94]. Umair et al. (2022) observed an increase in the TPC of carrot juice due to the combined effect of HVACP and ultra-high hydrostatic pressure (UHHP). According to the authors, the increase in TPC by plasma-induced active species was related to the disruption of cell membrane bonds and the biosynthesis of phenolic compounds through the metabolism of phenylpropanoid enzymes [111]. An investigation by Kashfi et al. (2020) indicated that treatment of peppermint with low-pressure cold plasma (LPCP) (20 and 50 W, for 20 min) increased the TPC in the extract compared with the control sample [109].

Keshavarzi et al. (2020) evaluated the efficacy of DBD cold plasma variables (gas, time, and power) on the TPC extraction rate from green tea leaves. They reported that the TPC of the samples decreased and increased after air and nitrogen DBD plasma treatment, respectively. In the first case, oxygen radicals formed during treatment with air CP led to the degradation of phenolic compounds. In contrast, in nitrogen CP treatment, ion bombardment led to erosion of the superficial epidermal layer of the leaves, facilitating solvent penetration and the extraction of more phenolic compounds. The study also showed that the interaction between time and power required an increase in both parameters to achieve the best TPC. Finally, the analysis of the individual phenolic compounds under optimal nitrogen CP conditions (15 W and 15 min) showed an increase in the concentration of gallic acid and catechin, with a slight decrease in epicatechin and epigallocatechin gallate (EGCG) [21].

Pogorzelska-Nowicka et al. (2021) evaluated the effect of CP pretreatment on the TPC of 12 types of grounded and water-suspended herbs. This approach has not been found in previous studies. In 11 extracts from 12 herbs, they observed a significant increase in total phenols (approximately 10%) after the CP treatment (8 min, 20 kHz). This was probably due to the destruction of the cell membrane by reactive plasma species. In addition, the hydration of ground herbs can facilitate the extraction process because of the equal penetration of radicals in the whole sample surface. They also stated that the breakdown of larger polyphenols into minor compounds can increase the TPC of the extract. Anthocyanin and flavonoid content also increased but only in four herb extracts. However, in 8 out of the 12 samples, no significant difference was observed in individual phenolic compounds [19]. Rodríguez et al. (2017) reported that nitrogen-CP treatment significantly increased the TPC of cashew apple juice. However, in the case of treatment performed for 5, 10, and 15 min, no significant differences between the samples were observed [97]. Lee et al. (2023) studied the effect of surface dielectric barrier discharge (SDBD) on oat (*Avena sativa* L.) sprout extracts [117]. In this study, oat seeds, after hydration for 12 h, were exposed to plasma for 6 min per day for 3 days after sowing, with no significant effect on sprout growth, but with a significant increase in the content of bioactive metabolites. They stated that the phenolic content of oat sprouts increased through the stimulation of the antioxidant system, the release of phenolic compounds, and their decomposition into minor compounds, similar to the study conducted by Kim et al. (2014) [108]. According to the results reported by the authors, the single plasma treatment (1 day) produced a significant increase in the content of free amino acids (39.4%), γ-aminobutyric acid (53%), and avenacoside B (23%) compared with the control, while the increase in the number of CP treatments (3-days) increased the content of hexacosanol, the most plentiful polycosanol found in oat sprouts [117]. Herceg et al. (2016) demonstrated that processing time and gas flow rate were able to improve the TPC of pomegranate juice [98].

Several researchers have also reported that CP treatment/pretreatment may reduce TPC. Faria et al. (2020) investigated the effect of glow discharge plasma (GDP) pretreatment on the ultrasound-assisted extraction of total phenolic compounds from sea asparagus. CP pretreatment for 60 min resulted in a significant decrease (83%) in TPC [20]. Afshar et al. (2021) investigated the effect of oxygen and nitrogen CP treatment on the physicochemical properties of oil extracted from sunflower and sesame seeds. They stated that the TPC in both extracted oils decreased significantly as exposure time increased, especially when an oxygen atmosphere was applied. Indeed, ROS in an oxygen-CP atmosphere can interact with phenolic compounds, leading to structural degradation that results in a decrease in total phenolic compounds [114]. Hemmati et al. (2021) reported that the total phenolic content of green tea powder decreased with the increase in exposure voltage (20, 22, and 25 kV) and time (2, 4, 6, and 8 min). Similarly, the TPC of apple juice was also reduced with increasing cold plasma treatment time [99,140].

Zielinska et al. (2022) investigated the modification of okra pod cell wall polysaccharides and phytochemicals using cold plasma. CP treatment (5, 15, and 30 s) slightly reduced the TPC of okra pods by 5, 13, and 20%, respectively. The results indicated that treatment with CP destroyed the basic structures of phenolic compounds and that oxidation of these compounds, such as phenolic acids and their derivatives, reduced TPC [100,106].

Almeida et al. (2015) reported the effect of CAP and ozone on prebiotic orange juice. Orange juice can be affected by direct and indirect DBD CP treatment exposure. They observed that the TPC decreased with 70 kV plasma treatment applied for 15–60 s [101]. Liu et al. (2021) and Leite et al. (2021) found a similar effect of DBD CP on the reduction in TPC in kiwi turbid juice and cashew apple juice extraction [102,103]. In other studies, Abedelmaksoud et al. (2022) stated that the TPC of mango pulp increased with the application of dielectric barrier discharge plasma (DBDP) for up to 6 min and then decreased at 8 and 10 min. According to the authors, the increase in phenolic compounds was caused by plasma polymerization and phenylalanine ammonia-lyase activity, which caused the decomposition of cell wall polysaccharides and the release of conjugated phenolic compounds [104]. Furthermore, Yodpitak et al. (2019) reported that the phenolic content of DBD-treated brown rice (PGBR) reached its maximum value and increased during germination within 0.5 days, whereas in the control sample (GBR), germination peaked after 1.5 days and then decreased rapidly [105,107,122]. Noteworthily, Seelarat et al. (2023) reported that the TPC of white *Cordyceps militaris* blended by *Cordyceps militaris* through cold plasma jet (CPJ), increased as time did too, from 30 s to 90 s, and decreased when the treatment time reached 120 s [110]. These studies have shown that the quantity and quality of the phytochemical profiles in the extracts are mainly related to the interaction of CP with the treated plant matrix.

Among the operating parameters of the CP process, the processing time can play a crucial role in the reaction between plasma reactive species and phenolics, e.g., by increasing the oxidation of flavanols [90]. The impact of processing time also depends very much on the chemical structure of the individual bioactive compound being considered. For example, in the case of anthocyanins, short CP treatment facilitates their extraction by disrupting the cell vacuoles where these substances are enclosed. On the other hand, longer treatment times (>20 min) can lead to a loss of anthocyanins due to photodegradation by UV photons generated in the plasma, which transform these compounds into chalcones and thus into benzaldehyde and benzoic acid [141].

These reactions are also affected by the type of plasma, and more operating variables, such as the power or voltage applied, the type of feed gas, the target phenolic compound, and the food matrix. Generally, DBD CP using high voltages (>60 kV) decreases the phenolic content of plant extracts due to oxidation side reactions because of the generation of a high concentration of ROS and ozone in the plasma. However, when DBD CP is used at high voltages but low frequencies (50 Hz), an increase in the hydroxybenzoic acid content can be observed. It is important to note that plasma-side reactions are not just limited to oxidation ones; they also include hydrogenation, dehydrogenation, hydrolysis, and dehydration reactions, which may boost or reduce the phenolic content of the plant extract [98]. For a more in-depth look at the effect of CP on the chemical structure of individual phenols, we recommend reading the review by Kumar et al. (2023) [30]. Considering these considerations, it seems clear that after treatment with CP, the phytochemical profile of a plant extract may differ due to the different degrees of structural decomposition of the cells or different side reactions that may occur during the extraction process. Further studies are therefore needed to determine the optimal CP processing conditions to maximize the phenolic compound yield of the extract and optimize its phytochemical profile. Table 3 summarizes the changes in phytochemicals induced by CP application.

### 5.2. Antioxidant Capacity

Antioxidants are key substances that can control free radicals, bind oxygen, and block oxidation, maintaining the nutritional and nutraceutical value of food [142]. Among the bioactive substances present in food, polyphenols are considered to contribute most to the antioxidant potential of foods and extracts of plant origin. Any chemical changes in the profile of these compounds, such as oxidative degradation and the cleavage of double bonds by reactive oxygen species generated through CP treatment, such as ${OH}^{.}$, $O^{3}$, and $O_{2}^{-},$ may increase or decrease the antioxidant capacity of the extract [77,92,143].

Several studies have therefore analyzed the effect of cold plasma on the antioxidant capacity of extracts and essential oils (EO) [144]. For example, Bao et al. (2020) reported an improvement in the antioxidant potential of the phenolic extract from tomato pomace when samples were subjected to HVACP pretreatment produced with different gases. Samples treated with nitrogen CP showed the highest improvement in antioxidant capacity (30%) compared with samples treated with argon, helium, and air plasma. They stated that this observed general improvement was probably related to the higher levels of more potent phenolic antioxidants and hydrophilic phenols present in the final extract due to the increase in surface hydrophilicity following the treatment [88]. Similarly, Poomanee et al. (2021) indicated that Luem Pua (LP) pre-treated with CP showed the highest increase in antioxidant capacity compared with the other tested species and untreated LP. The IC_50_ value recorded for the pretreated LP sample was 0.080 ± 0.002 mg/mL, while the value for the untreated LP was 0.187 ± 0.025 mg/mL. According to the authors, this improvement may result from the higher concentration of cyanidin-3-O-glucoside (CNG) and phenolic compounds in the pretreated LP extract, as no other new compounds were observed in the HPLC analysis to which this improvement could be attributed [122].

However, Bao et al. (2020) believed that another reason may be responsible for the improved antioxidant potential in plasma-treated samples. Indeed, they showed that when exposing the grape pomace to HVACP pretreatment, the phenolic extract obtained had a higher antioxidant potential regardless of the treatment duration (5, 10, and 15 min) than the untreated sample, due to the release of the intercellular compounds caused by the strong breakdown of the plant tissue following the experimental treatment. However, regardless of the overall increase in antioxidant capacity, it was observed a slight, though not significant, reduction in it from 5 to 10 min [94].

Interestingly, some researchers observed that although CP treatment reduces TPC, total flavan content (TFC), or vitamin C, the antioxidant potential remains unaffected. The possible reason for this stability is that the unbounded antioxidant compounds, which are released from carbohydrate and protein complexes during CP treatment, can be replaced by part of the antioxidant compounds that are decomposed by the plasma reactive species generated during CP treatment [96,100].

For instance, Faria et al. (2020) reported that despite the drastic loss of phenolic content in sea asparagus (*Salicornia neei*) extract, when the pre-treatment time with CP was extended to 60 min, the antioxidant capacity did not change significantly. One possible reason for this stability, according to the authors, could be the presence of more active antioxidants formed by highly reactive species by the CP pretreatment employed before ultrasound-assisted extraction (UAE). Notably, with a shorter CP exposure time (5 min), an improvement in the antioxidant capacity of the extract was observed, probably due to a more severe cell wall cleavage that led to greater solvent penetration [20].

Hou et al. (2019) also mentioned the importance of CP treatment exposure time since the antioxidant potential of blueberry juice (assessed by FRAP, ABTS, and DPPH techniques) declined when the operation time was extended from 2 to 6 min [145]. Similar results were reported by Abedelmaksoud et al. (2022) for fresh mango pulp treated with DBDP for four minutes [104].

It is worth noting that CP treatment does not always positively affect antioxidant capacity. Keshavarzi et al. (2020) indicated that air-working gas in CP pretreatment before the extraction of phenols from green tea leaves led to a significant reduction in the antioxidant capacity in all treatments evaluated [21]. According to the authors, ROS formed through air-CP might be responsible for the cleavage of phenolic compounds, thus reducing the antioxidant potential of the extracts. In contrast, cold nitrogen plasma increased the antioxidant potential. This increase can be due to the presence of specific phenolic compounds, usually restricted to the membrane and cell wall, which are now rapidly released as sufficient energy is supplied under CP conditions. Another potential reason can be the accelerated solvent permeability due to the severe tissue breakdown caused by ion bombardment during CP pretreatment. This investigation continued by optimizing nitrogen CP pretreatment conditions (exposure time of 15 min and generation power of 15 W) to reach the highest antioxidant capacity of the final extract (152.114 μM Trolox/g) by central composite design [21].

Kashfi et al. (2020) reported that plasma power values (20, 50, and 60 W for 20 min) during radiofrequency LPCP treatment affected the antioxidant potential of plant extracts. The authors showed that when peppermint (*Mentha piperita* L.) was subjected to LPCP immediately before the drying process, an increase in the antioxidant capacity of the extract was observed at a plasma power value of 50 W (IC_50_: 0.240 mg/mL), probably resulting from an increase in the concentration of bioactive compounds in the peppermint extract pretreated with plasma compared with the control sample. However, by increasing the power to 60 W, a significant decrease in the antioxidant capacity was observed. Furthermore, all the LPCP-treated samples demonstrated higher ferric-reducing ability compared with the untreated control sample [109].

In agreement with this study, Kim et al. (2019) pointed out that the intensity of active species generated during CP treatment was highly dependent on different CP parameters such as exposure time, generating power, plasma-working gas, and plasma source [120]. Pogorzelska-Nowicka et al. (2021) reported that, when subjecting various herbs (12 species) to cold plasma pre-treatment, the extract obtained from nine species showed a significant increase in their antioxidant potential, while *Sanguisorba officinalis* showed a slight reduction, and the extracts of *Andrographis paniculata* and *Polygonum aviculare* showed no significant changes following CP treatment [19]. As far as the effect of CP on the biological properties of essential oils (EOs) is concerned, Buonopane et al. (2019) reported that the EOs from CP-treated sweet basil plants showed a higher antioxidant capacity than untreated samples due to the higher eugenol extraction (48–94.82%) [121]. In contrast, Afshar et al. (2022) reported that for sunflower and sesame seeds pre-treated with CP, there was a significant decrease in the antioxidant capacity of the extracted oils, attributable to the degradation of phytochemicals during treatment, especially in cold air plasma, caused by the presence of ROS in the plasma. Furthermore, sunflower oil samples showed a higher scavenging capacity than sesame oil samples in the DPPH assay [114]. In this regard, it is worth noting that employing different methods provides a more accurate view of the potential antioxidant alteration in food products [97,146]. Several studies have shown different trends in antioxidant potential values about the type of in vitro test used to measure it, emphasizing the need to use more than one analytical test to more reliably determine the antioxidant potential of an extract or food, also depending on the fact that the antioxidant compounds most reactive to a specific type of method may be affected differently during plasma processing [96,145].

In agreement with the above statements, Ahmadian et al. (2023) reported inconsistent results when examining the antioxidant potential of extracts from hyssop (*Hyssopus officinalis* L.) pretreated with CAP using FRAP and DPPH techniques, respectively. According to the FRAP assay, pretreated samples with N_2_ and air plasma showed an increasing trend in antioxidant properties compared with control samples. In contrast, the DPPH assay showed that CAP treatment led to lower antioxidant capacity. However, N_2_ plasma-treated samples exhibited a higher radical scavenging rate than the untreated ones [96].

It is worth emphasizing that the different process parameters of the CP treatment, influencing the phytochemical profile of the extract, can similarly modulate its antioxidant potential. Rodríguez et al. (2017) reported that among different process parameters during the CP treatment of cashew apple juice, N_2_ plasma flow rate and exposure time played a determinant role in the FRAP assay. The most significant changes were observed at the 15th min of treatment and flow rates of 30 mL/min (164% relative antioxidant activity) and 50 mL/min (163% relative antioxidant activity). In addition, the results showed that the DPPH and ABTS assays were significantly dependent on the period of plasma treatment of the sample, where a longer exposure time, regardless of the rate of N_2_ flow, resulted in a continuous decrease in the antioxidant capacity (the highest antioxidant potential was recorded at the 5th min of CP treatment) [97].

Almeida et al. (2015) considered the ABTS assay a more accurate method in comparison with the DPPH one to reflect the antioxidant capacity alteration, since the CAP-treated prebiotic orange juice showed lower antioxidant activity in ABTS technique, while no significant difference was observed among treated and untreated samples through DPPH assay [101]. Ramazzina et al. (2016) evaluated some functional properties of minimally processed Pink Lady apples treated with DBD CP in comparison with untreated samples, through different in vitro and ex vivo tests, pointing out that plasma treatment caused only a slight reduction in antioxidant content and antioxidant capacity (up to 10%), mainly limited to the amphiphilic fraction [147].

It is worth noting that CP treatment may reduce antioxidant properties or even have no significant effect, due to the oxygen in the air plasma, which plays a role in suppressing antioxidant capacity by creating highly destructive ROS. Regardless of different types of working gases, the CP process parameters such as exposure time, power, and storage period have an important role in the antioxidant capacity changes. However, further investigation is recommended for a more accurate determination of different action mechanisms of the CP treatment on the antioxidant, through simultaneous assays such as ABTS, FRAP, and ORAC, to gain more reliable information. An overview of the effects of CP on antioxidant capacity is shown in Table 3.

### 5.3. Antimicrobial Properties

The use of plant extracts for the control of microbial infections and food spoilage is becoming increasingly popular in recent years. The ability to eliminate or control an exhaustive range of harmful organisms by complex plant extracts or essential oils, rich in polyphenols, terpenoids, and alkaloids, has led to the use of these bioactive compounds as food preservatives [148,149]. In this regard, the quality of the extraction process is one of the essential factors affecting the antimicrobial properties of plant extracts. In some studies, CP has been introduced as a pioneering technique for obtaining a high-quality extraction process with minimal loss of valuable compounds, thus preserving their antimicrobial properties. In one of these studies by Pogorzelska-Nowicka and coworkers (2021), the antibacterial potential of various dried herb extracts obtained by pretreatment with CP was evaluated against aerobic bacteria. Overall, the CP samples reduced total aerobic bacteria and most of them showed germicidal properties. Depending on herb species, the number of aerobic bacteria decreased by 1.27 up to 3.48 logCFU/g [19].

Kashfi et al. (2020) evaluated in vivo the efficacy of dried peppermint (*Mentha piperita* L.) extracts obtained by LPCP at three different powers (20, 50, and 60 W) for 20 min against the pathogen *E. coli* [109]. According to experimental observations, the bacterial growth was completely controlled with peppermint extract using high CP powers (50 and 60 W), whereas this process was unable to inhibit *E. coli* at 20 W. Faria et al. (2020) investigated the effect of ultrasound-assisted CP pretreatment on in vitro antimicrobial activity of the *S. neei* extract against *S. aureus* and *E. coli* by the disc diffusion method [20]. The *S. neei* extract (0.1 g/mL) showed an inhibition halo (4 mm) against *E. coli* and no inhibition halo against *S. aureus*. In general, the decomposition of the extract caused by CP treatment produces bioactive compounds such as ferulic, caffeic, and *p*-coumaric acids, which have shown synergistic antibacterial properties. Despite the few studies on the indirect antimicrobial effects of CP through the use of the plant extract obtained by this technique, many results confirm the direct application of CP in food disinfection.

Most studies stated that the microbial count decreased while increasing plasma treatment time and voltage [99,108]. In general, it has been found that Gram-negative bacteria are more sensitive to plasma treatment than Gram-positive ones. CP efficacy seems to be directly correlated to bacterial cell wall thickness. Gram-negative bacteria are characterized by a thin (<10 nm) inner cell wall layer and an additional outer membrane, consisting of phospholipids and lipopolysaccharides that are highly sensitive to plasma-generated ROS. Moreover, due to the presence of porins on the outer membrane of these bacteria, plasma penetration is certainly more effective than in the case of Gram-positive bacteria, which are characterized by a thicker peptidoglycan-based cell wall (20–80 nm) that cannot easily break down by reactive plasma species [150,151,152]. However, Hemmati et al. (2021) noticed that CAP was more effective in inhibiting Gram-positive bacteria (*E. faecalis*) than Gram-negative ones (*E. coli*) [153].

A summary of the effects of CP on the antimicrobial properties of the plant extracts obtained is given in Table 3. To date, studies on the antibacterial properties of these extracts are lacking, making it difficult to assess their efficacy. Therefore, it is necessary to extend research studies in this area with the help of statistical strategies to model and optimize the extraction conditions.

### 5.4. Other Relevant Biological Properties

The use of cold plasma to produce phytoextracts is a very recent technique, and most studies have focused on the effect of the extraction technique on the antioxidant and antimicrobial activity of the extracts obtained. However, some studies have evaluated other biological properties of phytoextracts obtained using plasma. Poomanee et al. (2021) optimized the CP extraction of some purple rice varieties, obtaining up to a five-fold higher amount of Cyanidin-3-O-glucoside in plasma-treated samples compared with the untreated one, with a significantly more substantial anti-ageing potential [122].

Mehta et al. (2022) optimized the extraction process of polyphenols by atmospheric and vacuum CP from rice and maize bran. The resulting phytoextracts showed not only higher antioxidant activity, but also better in vitro digestion, higher anti-inflammatory responses, and better overall quality than a conventional extraction [129]. In another study, they extracted xylooligosaccharides (XOS) from rice and corn bran dietary fibers by CP. Similar to polyphenols in their previous study, the extracted XOS showed better gastric digestion and anti-inflammatory responses with no cytotoxicity against RAW 264.7 and HepG2 cell lines [154].

Jeong et al. (2019) found that atmospheric DBD cold plasma treatment resulted in the dimerization of *trans*-resveratrol in a methanol solution. The generated *trans*-resveratrol dimers showed higher inhibitory activities against α-glucosidase and α-amylase than parent *trans*-resveratrol. Inhibiting the activity of these enzymes can contribute to the improvement of anti-diabetic properties through controlling postprandial hyperglycemia, and reducing the risk of developing diabetes [155]. The lack of studies on the biological properties, such as anticarcinogenic or anti-mutagenic, of plasma-derived phytoextracts, opens exciting new research prospects for scholars in the field to implement this technique commercially.

## 6. Conclusions and Future Perspective

Cold plasma is a new non-thermal technology that has shown considerable potential for food disinfection at low temperatures, with minimal energy consumption and cost. It has been introduced in recent years also as an advanced technique to produce high-quality phytoextracts with minimal side effects. The preservation of the main plant components and low impact on the internal matrix of the product is an essential advantage of CP compared with other traditional extraction methods. In the extraction industry, it represents an environmentally friendly process, with no production of toxic and hazardous waste compounds and no need to use water or solvents, which is a high added value in the preparation of high-quality plant extracts. However, like all nascent technologies, for its full exploitation at the industrial level, many studies are needed to design the system, its scalability, etc.

CP leads to an increase in extraction efficiency and, in general, improves the antioxidant properties of the extracts, which mainly depend on the phenolic compounds present in them. However, the complete control of CP treatment to achieve optimal extraction conditions is rather difficult due to the involvement of several process parameters that may enhance or suppress the antioxidant potential of the extracts. For the technique to take off, especially on a commercial scale, further investigations are therefore needed to in particular the mechanisms of plant cell wall disruption caused by CP and the interactions between phenolic compounds and plasma reactive species on a molecular or atomic level.

More studies are also needed in the food sector, where researchers have recently shared a new perspective on the ability of CP to reduce allergens in plant-derived foods. However, the mechanisms involved in this process are not entirely clear and require further investigation.

Studies in the literature have also highlighted the possible cytotoxic activity of plasma-treated solutions, which can be successfully used in the treatment of cancer cells, but parallel data on the effect these same solutions would have on healthy cell lines are lacking. In contrast, the results of studies on the mutagenic activity of plasma-treated protein solutions are mixed. Currently, the technique appears very promising for the preparation of phytoextracts, but for it to find industrial application, further studies are needed on the wholesomeness of the products obtained by this technique and thus on the interactions between the reactive species generated during plasma treatment and the primary and secondary metabolites present in the plant matrix.

## Figures and Tables

**Table 1:** Main phytochemical classes and related biological properties (adapted from [3]).

Phytochemical Class	Representative Compounds	Biological Properties
**1. Terpenoids**		
Monoterpenes	Pinene, Citronellol, Limonene	Aroma, Digestion, Antiseptic
Diterpenes	Gibberellic acid, Steviol	Aroma, Antioxidant
Triterpenes	Saponins, Glycosidic tri-terpenoids	UV protector, anti-inflammatory, healing, anti-edema activity
Tetraterpenes, Carotenoids	β-Carotene, Lycopene, Xanthophylls, Lutein	Anticancer, Antioxidant, Countering macular degeneration
**2. Polyphenols**		
**2.1. F** **lavonoids compounds**		
Flavonols Flavones Flavanones Flavan-3-ols Isoflavones	Quercetin, Myricetin, Kaempferol Luteolin, Apigenin, Hesperetin, Naringenin (+)-Catechin, (-)-Epicatechin, (-)-Epigallocatechin Genistein, Daidzein, Formononetin	Antioxidant, Anticancer, Astringent, Phytoestrogenic
**2.2. Non-flavonoid compounds**		
Lignans Stilbenes Tannins	Secoisolariciresinol Resveratrol Tannin acids	Photosensitization, Antioxidant, Astringent
**2.3. Phenolic Acids** Hydroxybenzoic acids Hydroxycinnamic acids	Gallic acid, Salicylic acid Ferulic acid, Coumaric acid, Caffeic acid	Antioxidant
**3. Sulfur-containing compounds**		
Thiosulfinates	Allicin	Aroma, Antiseptic, Anti-obesity, Anti-aging
Glucosinolates	Allylglucosinolate	Aroma, Thyroid hypertrophy, Inhibition of *Helicobacter pylori*
**4. Nitrogen-containing compounds**		
**Capsaicinoids**	Capsaicin	Burning taste, Analgesic
Betalains	Betacyanins, Betaxanthins	Color

**Table 2:** Comparison of different extraction methods for biologically active compounds.

Type of Extraction	Advantages	Disadvantages	Ref
Supercritical fluid extraction (SFE)	The solvent (CO_2_) is inexpensive and has low toxicity No solvent residue Possibility of recycling the solvent Pure extraction ratio Low-extraction temperatures Prevention of thermal damage Environmentally friendly	Destruction of the intended compounds using an unsuitable solvent Specialized high-cost equipment	[32,33,34,35,36,37]
Microwave-assisted extraction (MAE)	Laboratory and industrial scale Less time consumption High returns on investment	Low efficiency for non-polar compounds or viscous solvents Not suitable for heat-sensitive compounds Reduction in the yield of the phenolic components due to oxidation Expensive equipment and difficult to operate	[38,39,40,41,42,43]
Ultrasound-assisted extraction (UAE)	Large-scale commercial use Easy to handle Low solvent consumption Increase efficiency in a short time High extraction quality No need for complex instruments Relatively low-cost Short reaction/preparation time Use of a small amount of material	Attenuation of ultrasonic waves due to the presence of dispersed phase Low efficiency in extracting oils Not appropriate for heat-sensitive compounds Destructive effect on the active components of the plant by the formation of free radicals	[34,44,45,46,47,48,49]
Soxhlet extraction	Very simple to use Inexpensive Maintain the temperature in the system High extraction efficiency	High extraction time High consumption of solvent No agitation to speed up the process Decomposition of heat-sensitive ingredients	[47,50]
Hydro-distillation extraction	Inexpensive No need for organic solvent Widely used for the isolation of essential oils	It takes a long time to separate the solvent from the product No agitation to speed up the process Thermal decomposition High energy consumption	[51,52]
Steam- distillation extraction	Can extract water-insoluble compound	Low process speed	[53,54]
Solid phase extraction (SPE)	Rapid, economical, and sensitive technique Low cost and solvent consumption No wasting time High extraction efficiency Reduction in evaporation volumes High selectivity, more reproducibility Prevent emulsion formation Possibility of storage of enriched analytes on solid adsorbent	The difficulty of mastering the use and the possibility of developing the method The possibility of not completely removing existing interferences Sample extracts being insufficiently clean	[55,56,57,58,59,60]
Liquid-liquid extraction (LLE)	Simple operation Simple apparatus	Low selectivity Formation of emulsions that are difficult to break Difficult handling in large-volume samples A large volume of solvents (environmental pollution)	[61,62,63,64,65]
Cold plasma	Environmentally friendly process No production of toxic and hazardous waste compound Capable of operating at atmospheric pressure and ambient temperature Rapid sterilization The possibility of decontamination and high efficiency at low temperatures with minimal energy consumption and cost Inhibition and inactivation of surface microorganisms in a short time via the production of various reactive oxygen species (ROS) Low impact on the internal matrix of the product Application without water or solvents Efficient resource Accurate production of usable plasmas Removal of food volatile compounds Enzymes deactivate in solid and liquid food processing industries	Increasing the probability of lipid oxidation in high-fat foods Reduction in water holding capacity and water leakage during food storage (for example, fish fillets) Need for trained and experienced personnel Initial installation cost Security measures Need for special equipment Inability to deactivate internal enzymes in fruits Reducing the firmness of the fruit	[1,65,66,67,68,69,70,71]

**Table 3:** An overview of the effects of cold plasma on the extraction yield and quality of plant extracts.

Food Sample	Plasma Process			Extraction Efficiency	Main Results			Ref
	Type of Plasma	Extraction Condition	Application		Antioxidant Capacity	Phenolic Compounds	Antimicrobial Activity	
Tomato pomace	HVACP *	Gas: air, argon, helium, and nitrogen Voltage: 60 kV Time: 15 min	PC extraction	TPC extraction increased using He and N_2_ plasma by nearly 10%	Antioxidant potential increased by nitrogen CP (30%) in comparison with argon, helium, and air active gases.	No effect on trans-Ferulic acid, Gallic acid, Rutin and Isoquercetin Increase in Caffeic acid, Chlorogenic acid, Quercetin, and Naringenin	NA	[88]
Hyssop	CAP	Gas: air, nitrogen Voltage: 30 kV Frequency: 345 kHz Time: 5, 10, and 15 min	PC extraction	Increase in TPC extraction	FRAP: increased in both air and N_2_ plasma. DPPH: Decreased by air and increased by N_2_. The highest DPPH values were about 60.83 and 60.782% in nitrogen CP-pretreated samples for 5 and 15 min at a concentration of 1000 ppm, compared with un-pretreated sample prior to ultrasound-assisted extraction (DPPH: 77.939%) and conventional solvent extraction method (DPPH: 66.020%) at the same concentration.	NA	NA	[96]
Cashew apple juice	Cold Plasma (Nitrogen)	Gas: nitrogen Frequency: 80 kHz Time: 5, 10 and 15 min	Extract	TPC extraction increased using indirect nitrogen-cold plasma	FRAP: Increased by elevating N_2_ flow rate. The highest relative changes were about 164 and 163% after 15 min of treatment and flow rates of 30 and 50 mL/min. DPPH: Decreased by time extending. The highest antioxidant activity was observed in samples with minimum treatment duration (5 min) and flow rate of 50 mL/min (increased about 130%).	NA	NA	[97]
Pomegranate juice	Jet plasma	Voltage: 2.5 kV, 4 W Frequency: 25 kHz Time: 3–7 min	PC extraction	TPC extraction increased to the 33.03% by plasma operating condition (treatment time 3, 5, 7 min; gas flow 0.75, 1, 1.25 dm^3^ min^−1^)	NA	Increase in Catechin, Ellagic acid, Protocatechuic acid, Ferulic acid and p-Cumaric acid Decrease in Gallic acid, Chlorogenic acid, Caffeic acid and Punicalagin	NA	[98]
Green tea powder	CAP	Voltage: 20, 22 and 25 kV, Time: 2, 4, 6 and 8 min	Extract	Decrease in TPC extraction	FRAP and DPPH: decreased as the voltage increased and the treatment period lengthened compared with the untreated powder.	Decrease and Increase in Caffeine after 22 and 25 kV for 8 min, respectively Decrease in Linalool content (except at 20 kV for 2 or 4 min) Decrease in Geraniol after 20 kV for 8 min treatment	0.97, 1.35, and 1.43 log reduction after 8 min and 25 kV exposure treatment in total microbial, mold, yeast, and coliform counts, respectively	[99]
Green tea leaves	DBD plasma	Time: 5 and 15 min Power: 5 to 15 W Frequency: 6 kHz Gas: Air and N_2_	PC extraction	TPC extraction decreased 16.83% and 33.29% by the air and increased 41.14% by nitrogen CP, respectively	Antioxidant capacity decreased by air active gas and increased by nitrogen CP pretreatment. Nitrogen DBD treatment increased the antioxidant activity by 41.06% at 15 min and 15 W compared with un-pretreated green tea leaves.	Slightly increase in Gallic acid Increase in Catechin Decrease in Epicatechin and EGCG	NA	[21]
Okra pod	Cold Plasma and Air Atmospheric Pressure Plasma Jet	Time: 5, 15 and 30 s Power: 750 W Frequency: 20 kHz Gas: Air and N_2_	PC extraction	5, 15, and 30 s treatment decreased TPC by 5, 13, and 20% compared with non-treated sample	FRAP: increased by 20% until 5th s then decreased by 3 and 7% after 15th and 30th s of CP treatment. ABTS: increased by 4% until 5th s then decreased by 6 and 8% after 15th and 30th s of CP treatment. DPPH: no significant increase until 5th s and then decreased slightly by 5 and 9% after 15th and 30th s of CP treatment.	NA	NA	[100]
Orange juice	DBD-ACP	Time: 15, 30, 45 and 60 s Power: 70 kV Frequency: 50 Hz Gas: Air and N_2_	PC pretreatment	Decrease in TPC from 2.52 g/L in control sample (without ozone or plasma treatment) to 2.37 and 1.93 g/L for direct and indirect exposure, respectively	ABTS: decreased about 50% after 60th s of direct exposure to plasma field. DPPH: no significant difference.	NA	NA	[101]
Water-Suspended Herbs	Plasma jet system	Activated by nitrogen plasma for 8 min at 20 kHz	Extract extraction	TPC extraction increased using N_2_ CP treatment in 11 herb extracts up to 77% compared with untreated extracts gained from non-treated water suspended herbs at 70° C for 10 min	Antioxidant potential increased and decreased in 9 and 1 herbs extracts, respectively. Maximum increase was observed in *Vaccinium myrtillus* (DPPH; 0.567, FRAP: 0.404 and ABTS: 0.596 M ascorbic acid/g dw). Lowest antioxidant activity was observed in *Urtica dioica* (DPPH: 0.005 M ascorbic acid/g dw) and *Leonurus cardiaca* (FRAP: 0.021 and ABTS: 0.054 M ascorbic acid/g dw).	Increase in Anthocyanins and Flavonoids content only in four herb extracts	2.58 log CFU/g reduction in aerobic bacteria	[19]
Kiwi turbid juice	DBD	Voltage: 13, 22, 31 W; 15, 25, 35 kV Frequency: 60 Hz Time: 1–5 min	PC extraction	Decrease in TPC extraction	NA	NA	NA	[102]
Cashew apple juice	DBD	Voltage: 20 kV Frequency: 200, 700 Hz Time: 15 min	Extract	Decrease in TPC	NA	NA	NA	[103]
Mango Pulp	DBDP	Voltage: 25 kV Time: 0, 2, 4, 6, 8 and 10 min	Extract	Increase in TPC up to 6 min treatment time (19.39 mg GAE/100 mL) then reduced (16.43 mg GAE/100 mL) in comparison with untreated fresh extracted mango pulp (18.28 GAE/100 mL)	DPPH capacity percentage increased until 4th min of operation (about 7.9%) but then decreased by 3.4% until 10th min of operation.	NA	NA	[104]
Germinated brown rice (GBR)	DBD	Voltage: 100, 135, 170 and 200 W Time: 25–300 s	PC extraction	Increase in TPC in cold plasma-treated GBR from approximately 30% to 86% compared with un-pretreated germinated brown rice	No significant differences observed in all rice cultivars and control samples through DPPH assay.	Increase in Anthocyanins and Phenolic content	NA	[105]
Wolfberry	Cold Plasma and Air Atmospheric Pressure	Voltage: 750 V Frequency: 20 kHz Time: 15–60 s	Extract extraction	Decrease in TPC from 17.1 to 13.5 mg GAE/g DM compared with untreated sample (30 and 45 s had the highest value)	NA	NA	NA	[106]
Sea asparagus	GDP	Time: 5 and 60 min Power: 14 W	Extract extraction	NA	Antioxidant capacity increased after 5 min CP treatment.	NA	Reduction in *E. coli* No effect on *S. aureus*	[20]
Grape pomace	HVACP	Voltage: 60 kV Time: 5, 10, and 15 min	PC extraction	TPC extraction increased after CP treatment (5 and 15 min) by 10.9–22.8% compared with un- HVACP treated grape pomace	Increase by about 29.0 and 34.7% after 5 and 15 min treatment, along with a slight decrease in antioxidant capacity from 5 min to 10 min.	Increase in Quercetin Increase in Anthocyanins and Anthocyanidins content (after 5 and 15 min treatment)	NA	[94]
Lotus Petal Powder	DBD	Voltage: 0–11 kV Frequency: 50 Hz Time: 5 min	Extract	Increase in TPC by 5.37% compared with microwave dried LPP without CP treatment	FRAP: increased from 42.92% (Control sample:) to 76.63% (CP-treated LPP).	Increase in Nuciferine (7.07–8.85%), Pronuciferine (0.86–1.58%), α-Amyrin (1.03–7.36%) and β-Amyrin (0.62–4.64%) Decrease in Tetracosanoland (53.44–29.05%) and Phthalic acid (0.73–0.14%)	NA	[107]
Naringin	DBD	Power: 250 W Frequency: 15 kHz Time: 0–20 min	Naringin treatment	Increase in TPC from 172.50 to 225.83 ppm in comparison with untreated sample	DPPH: increased about 36.75% by 20 min plasma treatment. FRAP: a time-dependent increase.	NA	Decreased in *S. Typhimurium, L. monocytogenes, S. aureus*, and *E. coli O157:H7* after 20 min treatment	[108]
Peppermint	LPCP	Power: 20, 50 and 60 W for 20 min	Extract	Increase in TPC from 262.81 to 292.53 mg GAE/g edw at 20 and 50 W then reduced at 60 W (255.28 mg GAE/g edw)	DPPH: increased by elevating the power to 50 W (Up to 83%) and then with using 60 W of power, decreased to the initial value of untreated peppermint powder as the control sample (74%).	NA	Reduction in *E. coli* after 50 and 60 W treatments	[109]
Blended scarlet caterpillarclub	CPJ	Voltage:12 kV Frequency: 25 kHz Time: 30–120 s	Extract extraction	Increase in TPC extraction compared with the Untreated blended *C. militaris*	ABTS: highest increase at 60 and 90 s (6.82% and 6.54%) then decreased in 120 s (9.08%). DPPH: After 60 to 120 s of treatment the antioxidant activity enhanced by 12 to 17% and the highest observed in 90 s. FRAP: After 60 s exhibited the highest increase (50.35%) and then decreased after 120 s (26.46%).	NA	NA	[110]
Carrot juice	HVACP	Voltage:70 kV Time: 3 min	Extract extraction	Increase in TPC from 9.15 to 11.89 μg/g by HVACP-UHP treatment	NA	NA	NA	[111]
Fenugreek (dried and soaked seeds)	HVACP	Frequency: 80 kHz Time: 30 min	Galactomannan extraction	Increase in extraction yield (sample DS: 67%, sample SS: 122%)	NA	NA	NA	[112]
*Camelina seeds*	DBD-Plasma	Voltage: 15, 18, and 21 kV Time: 2,4,8 and 16 min	Oil extraction	Increase in extraction yield (from 24.3 to 31.5%)	NA	NA	NA	[113]
Sesame Sunflower. seeds	DBD-Plasma	Voltage: 25 kV Frequency: 50 Hz Time: 0–30 min Gas: O_2_ and N_2_	Oil extraction	Increase in extraction yield in comparison with the untreated control samples without storage time	Extending the treatment time and storage duration (from 0 to 30 min and 0–30 days) led to a significant decrease (25%) in the antioxidant potential of both oil types.	NA	NA	[114]
Fennel seed Spearmint leaf	DBD-Plasma	Voltage: 17–23 kV Time: 5–15 min	Essential oil extraction	Yield extraction increased after CP treatments at 15 kV for 5 min (1.83% *v*/*w*) and decreased after 23 kV for 17 min (1.81% *v*/*w*) compared with untreated sample	NA	NA	NA	[115]
Lemon peel	DBD-Plasma	Voltage: 1–11 kV Frequency: 50 kHz Gas: O_2_ and N_2_	Oil extraction	Increase in extraction yield (149.34%) compared with microwave extraction method	NA	NA	NA	[116]
Oat sprout	SDBD	Frequency: 14.4 kHz Voltage:8 kVp Power: 51.7 W Time: 6 min/day for 3 days	Extract	Increase in TPC (8.5% higher than untreated control)	NA	Increase in hexacosanol (28% higher than untreated sample)	NA	[117]
Lemon verbena leaves	LPCP	Frequency: 50 kHz Gas: nitrogen (N_2_), argon (Ar), and oxygen (O_2_)	Essential oil extraction	Increase in anthocyanin extraction yield	NA	NA	NA	[118]
Lemon peel	DBD-Plasma	Frequency: 50 kHz Voltage: 30 kV Gas: nitrogen (N_2_), argon (Ar), and oxygen (O_2_) Time: 1–15 min	Essential oil extraction	Increase in extraction yield	NA	NA	NA	[119]
Prickly Pear Cactus Fruit	LPCP	Frequency: 2.45 GHz of microwave Time: 10 to 40 min Power: 600–900 W	CP treatment on extract	NA	The highest increase occurred at 40 min with a power setting of 750 W, resulting in a 1.77% increase. Conversely, the greatest decrease was observed at 25 min also with a power setting of 750 W, resulting in a decrease of about 4.01%. It is important to note that the untreated powdered Prickly Pear Cactus fruit was used as the control sample for comparison.	NA	NA	[120]
Sweet basil	Cold Plasma and Air/Helium Atmospheric Pressure Plasma Jet	Frequency: 1 kHz Time: 30 s Gas: air and helium	Seed pre treatment (Essential oil)	NA	EOs extracted from homegrown CP-treated Sweet basil seeds, demonstrated higher antioxidant capacity (81.55%) compared with the untreated conventional forms (46.36%) and commercial BHT (about 77.24%) at a concentration of 50 µg/mL. The antioxidant potential of CP-treated samples varied from 48% to 94.82% by increasing the concentration.	NA	NA	[121]
Purple glutinous rice	LPCP	Frequency: 13.56 MHz Time: 20 min Power: 100 W Pressure: 6 Torr	PC pretreatment	Increase in TPC (Luem Pua rice extract had the highest TPC)	The Luem Pua treated with CP (IC_50_ = 0.080 mg/mL) showed an increased antioxidant capacity compared with the untreated Luem Pua (IC_50_ = 0.187 mg/mL).	Increase in Anthocyanins and Flavonoids content especially in Luem Pua rice extracts	NA	[122]

* HVACP: high voltage atmospheric cold plasma; TPC: total phenolic content; PC: phenolic compounds; DBD: dielectric barrier discharge; GDP: glow discharge plasma; EGCG: epigallocatechin gallate; LPCP: low-pressure cold plasma; NA: not available.

## Supplementary Materials

The following supporting information can be downloaded at: https://www.mdpi.com/article/10.3390/foods12173181/s1, Figure S1a: Documents by year; Figure S1b: Documents by type; Figure S1c: Documents by subject area; Figure S1d: Documents by country or territory; Reprinted with permission from Scopus^®^. 2023, Elsevier. Table S1. Cluster composition of keyword co-occurrence network analysis from the cleaned dataset after SCOPUS search: “*cold plasma*” AND “*extraction*”. Descending order of total number of occurrences for each keyword in the cluster.

## List of Abbreviations

**Abbreviation**	**Definition**
CP	Cold plasma
CARG	Compound Annual Growth Rate
SFE	Supercritical fluid extraction
MAE	Microwave-assisted extraction
UAE	Ultrasound-assisted extraction
SPE	Solid-phase extraction
LLE	Liquid-liquid extraction
ROS	Reactive oxygen species
DBD	Dielectric barrier discharge
HVACP	High voltage atmospheric cold plasma
LPCP	Low-pressure cold plasma
RNS	Reactive nitrogen species
TPC	Total polyphenol content
LC-MS	Liquid chromatography-mass spectroscopy
UPLC	Ultra-performance liquid chromatography
EGCG	Epigallocatechin gallate
SDBD	Surface dielectric barrier discharge
GDP	Glow discharge plasma
PGBR	Phenolic content of DBD-treated brown rice
CPJ	Cold plasma jet
CNG	Cyanidin-3-O-glucoside
TFC	Total flavan content
DPPH	2,2-diphenyl-1-picrylhydrazyl
ABTS	2,2′-azino-bis(3-ethylbenzothiazoline-6-sulfonic acid
FRAP	Fluorescence Recovery After Photobleaching
ORAC	Oxygen radical absorbance capacity
RSM	Response surface methodology
ANN	Artificial neural network
*E. coli*	*Escherichia coli*
*S. neei*	*Salicornia neei*
*S. aureus*	*Staphylococcus aureus*
*E. faecalis*	*Enterococcus faecalis*
*S. Typhimurium*	*Salmonella typhimurium*
*L. monocytogenes*	*Listeria monocytogenes*
