# Supplementary material for: Cold Plasma-Assisted Extraction of Phytochemicals: A Review
Source: Foods. 2023 Aug 24;12(17):3181. doi: 10.3390/foods12173181 (PMC10486403; doi:10.3390/foods12173181)
Supplement: Supplementary file 1 [file foods-12-03181-s001.zip › Captions.pdf]

Captions

Supplementary Materials

Table S1. Cluster composition of keyword co-occurrence network analysis. Order of keyword listing in each cluster: number of occurrences.

Figure S1. (a) Trends in number of published documents per year (*Documents by year*). (b) Pie chart of the percentage of publications classified by type (*Documents by type*). (c) Pie chart of the percentage of documents classified by subject area (*Documents by subject area*). (d) Bar chart of top 10 countries in terms of absolute number of published documents (*Documents by country or territory*). Statistics of quantitative distribution of published documents in Scopus after the search (performed on 24th of April 2023): "*cold plasma*" AND "*extraction*" and subsequent sub-selection of the dataset. All the charts were generated with the *Analyse search results* tool in Scopus.
