# Supplementary material for: Cold Plasma-Assisted Extraction of Phytochemicals: A Review
Source: Foods. 2023 Aug 24;12(17):3181. doi: 10.3390/foods12173181 (PMC10486403; doi:10.3390/foods12173181)
Supplement: Supplementary file 1 [file foods-12-03181-s001.zip › TABLE_S1.pdf]

Table S1. Cluster composition of keyword co-occurrence network analysis from the cleaned dataset after SCOPUS search: "*cold plasma*" AND "*extraction*". Descending order of total number of occurrences for each keyword in the cluster.

Cluster Number (colour)	Nember of keywords	Keywords (Descending order of total number of occurrences)
1 (red)	14	cold plasma; extraction; cold plasmas; plasma applications; cold plasma treatment; plasma; scanning electron microscopy; animal; animals; dielectric materials; gas chromatography; mass spectrometry; nonhuman; solvent extraction
2 (green)	13	electric fields; ultrasound; anthocyanins; bioactive compounds; non-thermal processing; pulsed electric field; food handling; heat treatment; polyphenol; procedures; ultrasonics; hydrostatic pressure; oxidation
3 (light blue)	11	antioxidants; chemistry; plasma gas; plasma gases; antioxidant; antioxidant activity; polyphenols; phenols; phenolic compounds; plant extract; plant extracts
