# Supplementary figures and images for: Cold Plasma-Assisted Extraction of Phytochemicals: A Review
Source: Foods. 2023 Aug 24;12(17):3181. doi: 10.3390/foods12173181 (PMC10486403; doi:10.3390/foods12173181)

# Documents by year

Scopus

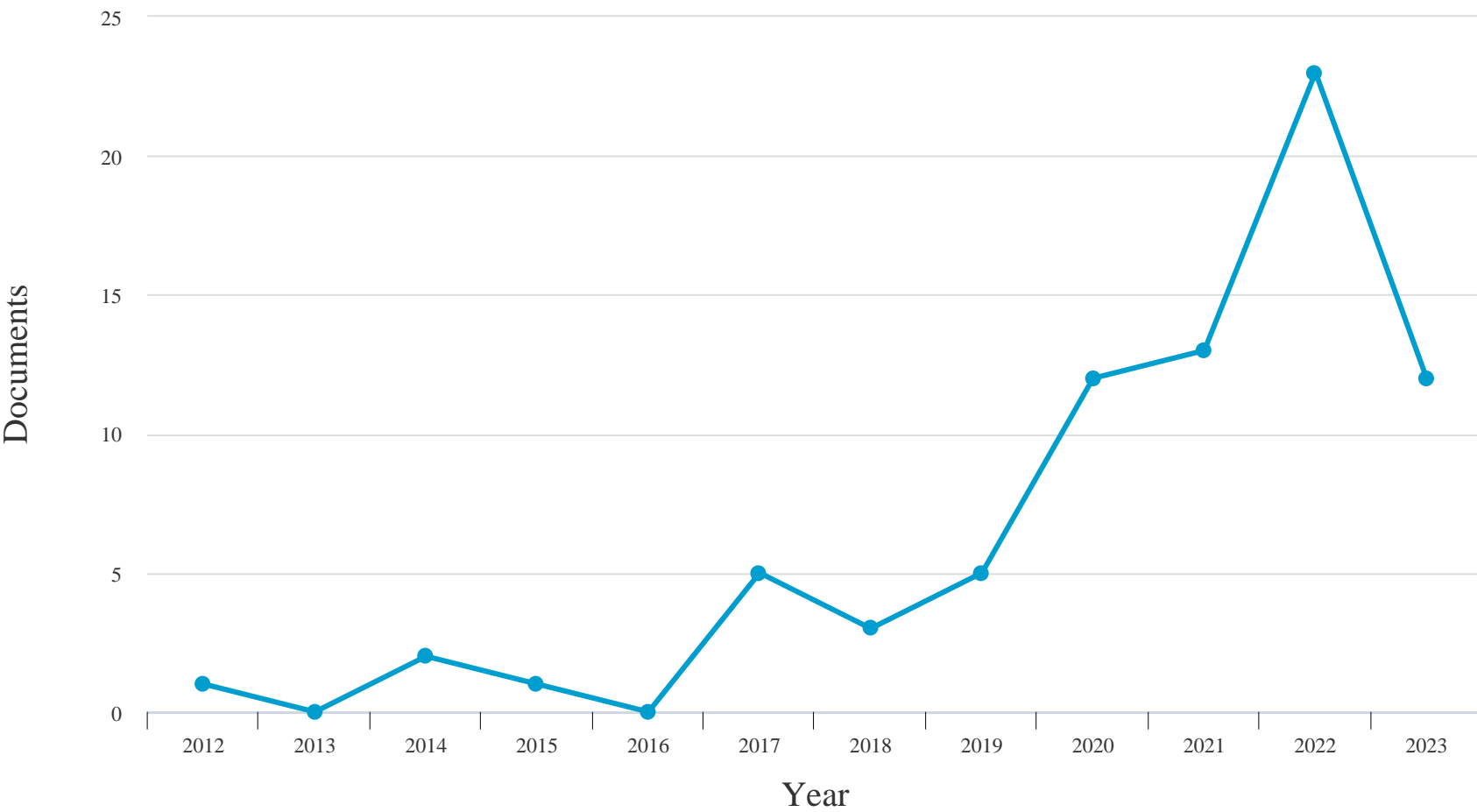

Supplement: Supplementary file 1 [file foods-12-03181-s001.zip › FIGS1_a.pdf]

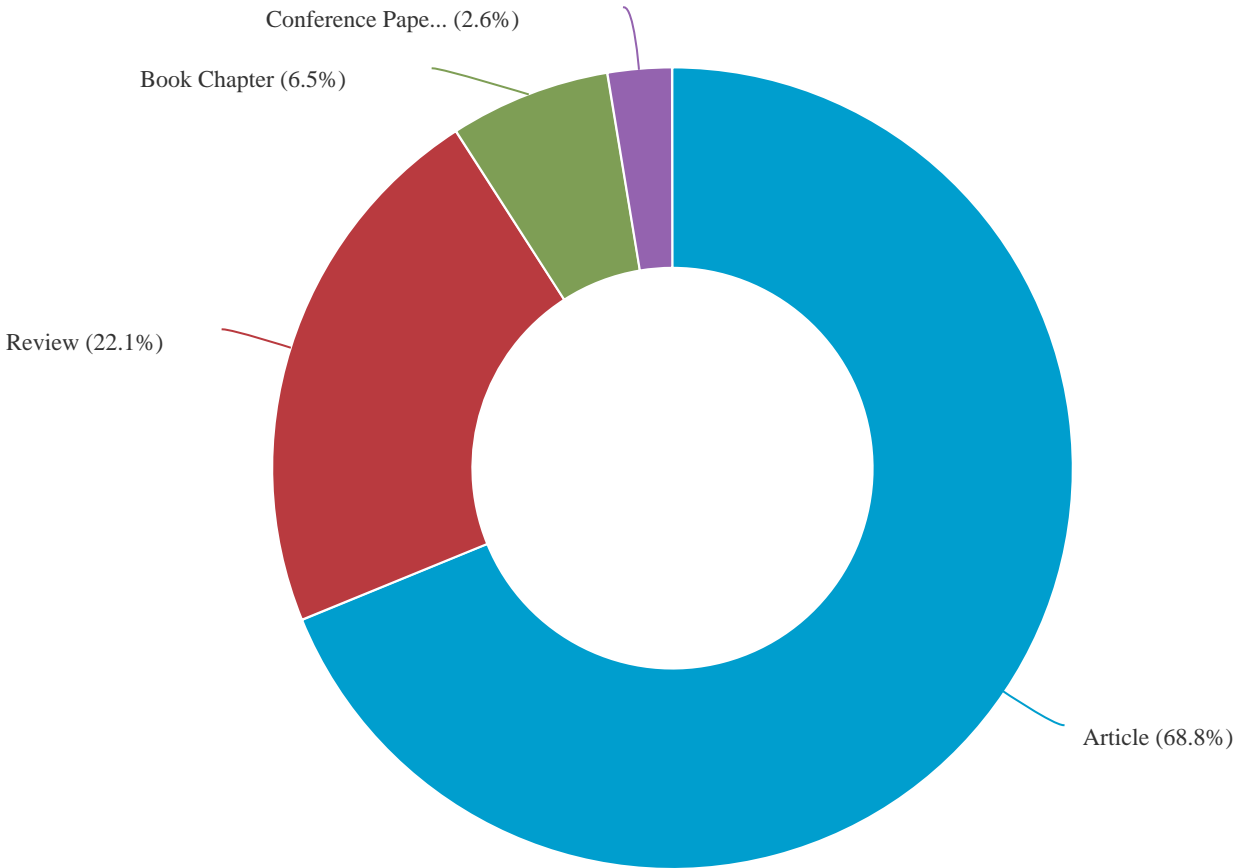

Supplement: Supplementary file 1 [file foods-12-03181-s001.zip › FIGS1_b.pdf]

# Documents by subject area

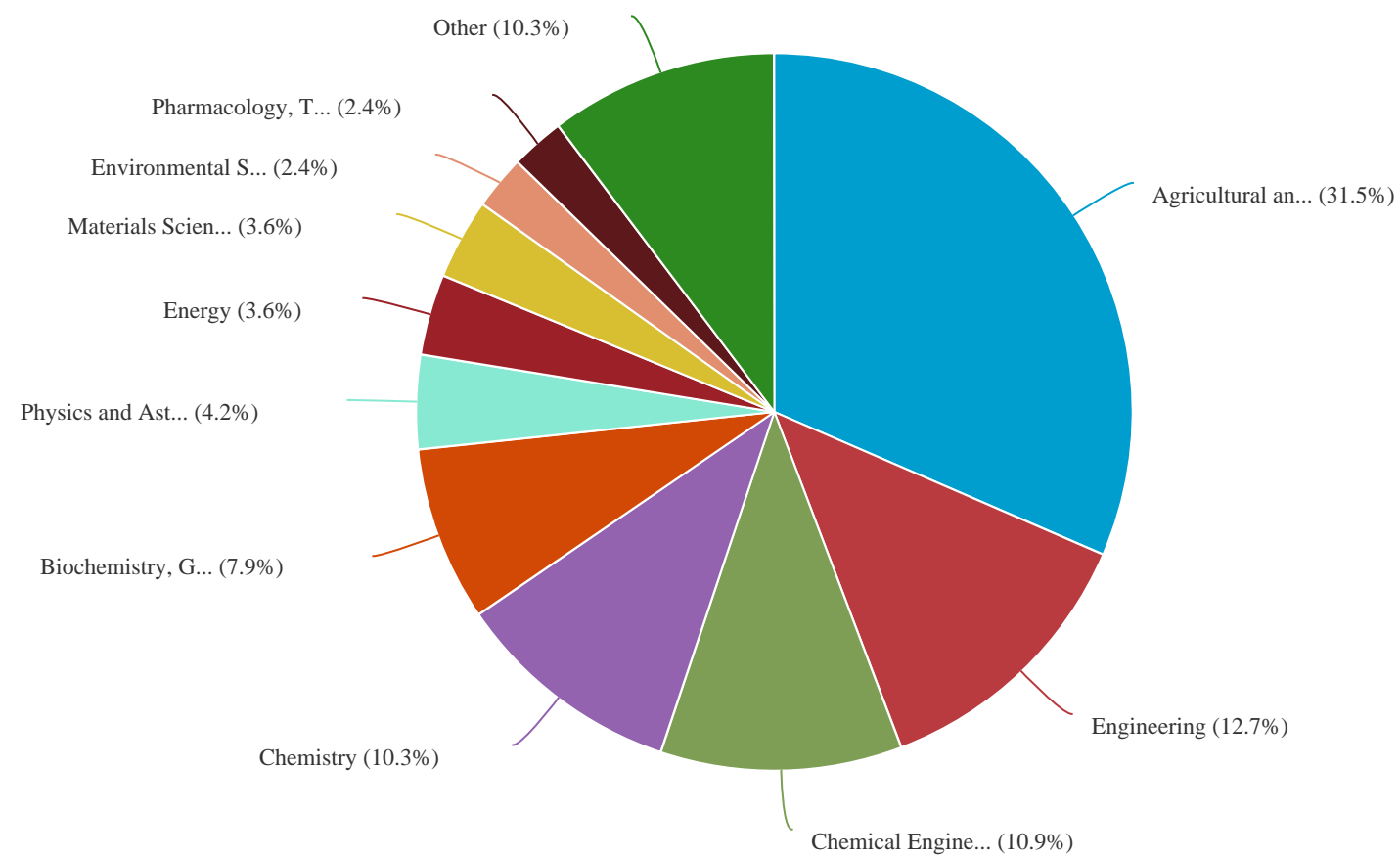

Supplement: Supplementary file 1 [file foods-12-03181-s001.zip › FIGS1_c.pdf]

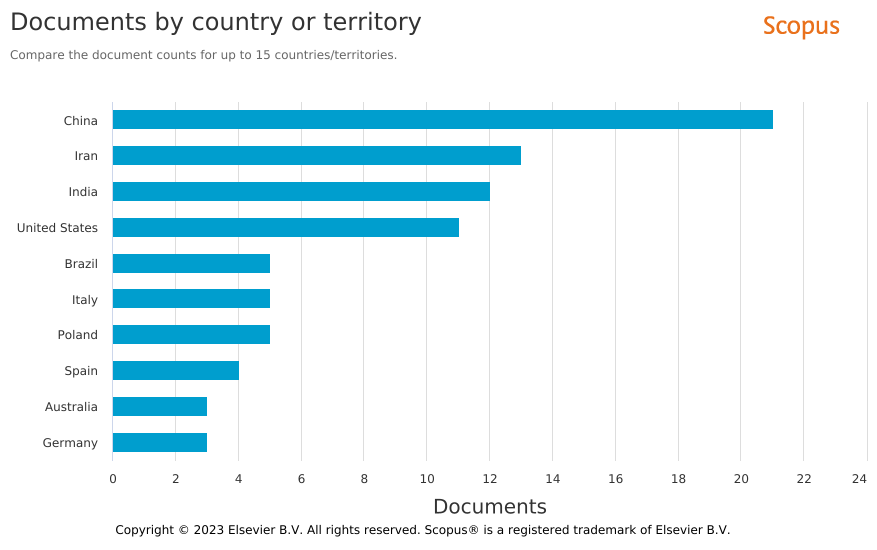

Supplement: Supplementary file 1 [file foods-12-03181-s001.zip › FIGS1_d.png]
